# Investigating the Chemical, Antifungal, and Cytotoxic Properties of *Cochlospermum vitifolium* Leaves and Stem Bark Extracts

**DOI:** 10.1002/cbdv.202503856

**Published:** 2026-04-24

**Authors:** Francisco A. S. D. Pinheiro, Addison R. Almeida, Matheus S. Menezes, Thomaz Rocha, Weslley S. Paiva, Hugo A. O. Rocha, Cristiane F. de Assis, Gislene Ganade, Laila S. Espindola, Leandro S. Ferreira

**Affiliations:** ^1^ Programa de Pós‐Graduação em Ciências Farmacêuticas, Departamento de Farmácia Universidade Federal do Rio Grande Do Norte (UFRN) Natal Brazil; ^2^ Departamento de Farmácia Universidade Federal do Rio Grande Do Norte (UFRN) Natal Brazil; ^3^ Programa de Pós‐Graduação em Ecologia, Departamento de Ecologia Centro de Biociências Universidade Federal do Rio Grande Do Norte (UFRN) Natal Brazil; ^4^ Laboratório de Biotecnologia de Polímeros Naturais‐BIOPOL, Departamento de Bioquímica Universidade Federal do Rio Grande Do Norte (UFRN) Natal Brazil; ^5^ Laboratório de Farmacognosia, Campus Universitário Darcy Ribeiro Universidade de Brasília Brasília Brazil

**Keywords:** antifungal, chemical profile, *Cochlospermum vitifolium*, cytotoxicity, leaves extract, stem bark extract

## Abstract

Comprehensive analysis of the chemical properties and bioactivities of plant extracts is essential for identifying compounds with therapeutic potential. This study analyzed hydroethanolic extracts from the leaves and stem bark of *Cochlospermum vitifolium*, investigating their chemical composition, antifungal, and cytotoxic activity. UHPLC‐MS/MS, GC‐MS and bioinformatic tools techniques were employed to characterize the chemical profiles. Antioxidant activities were evaluated using DPPH and ABTS assays, cytotoxicity assessed in L929 fibroblasts and MC3T3 osteoblast‐like cells, and antifungal activity was evaluated against *Candida* spp. The leaf extract exhibited higher total phenolic and flavonoid content, antioxidant capacity in both radical‐scavenging, and antifungal activity, reaching a minimum inhibitory concentration of 1.95 µg/mL against *Candida albicans*. None of the extracts showed cytotoxicity. PCA, chord diagram, and volcano plot indicated clear differences between the chemical profiles of the leaves and stem bark and supported the association between these metabolites and the observed antifungal activity. Finally, in vitro antifungal tests were performed for the first time with *C. vitifolium*, showing promising results against *Candida* spp. species, suggesting a safe alternative as an antifungal agent.

## Graphical abstract

This figure contains images created by Freepik from Flaticon.

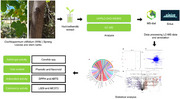

## 1 Introduction

There has been a significant rise in the global prevalence of fungal infections. Every year, millions of people are affected, with reports indicating that >600 fungal species are involved. The scope and severity of fungal infections can vary widely, ranging from superficial conditions that can be treated with topical medications to more severe cases, especially in immunocompromised patients [1, 2]. As the prevalence of fungal‐related diseases increases, urgent challenges have emerged, including a rise in reports of strains that are resistant to standard treatments. For instance, in hospital settings, *Candida glabrata* has become the most common fungus resistant to azole derivatives, frequently causing candidemia and other forms of invasive candidiasis [3].

The genus *Candida*, in the order *Saccharomycetales*, includes approximately 200 known species. Of these, around 20 can cause disease in humans, some of which act as opportunistic pathogens. *Candida albicans* is the most common pathogenic fungus found in humans. In addition, other species such as *C. glabrata*, *C. parapsilosis*, *C. tropicalis*, and *C. krusei* are frequently cited as causes of systemic candidiasis [4, 5].

Natural products are a diverse and essential source of bioactive molecules, with potential for the development of new drugs [6]. Various plant extracts, or compounds isolated from them, have demonstrated antifungal activity [7, 8]. This effectiveness has been observed even when combined with azoles, polyenes, or echinocandins to evaluate their synergistic effects in treating *Candida* spp. infections [9].

The genus *Cochlospermum*, with 19 species distributed globally, belongs to the Bixaceae family [10]. Species within this genus are used in traditional medicine across several Latin American countries, including Guatemala, Cuba, Mexico, and Panama, for the treatment of malaria, fevers, jaundice, diabetes, diarrhea, stomach disorders, typhoid fever, and urinary tract infections [11]. These plants are recognized for their diverse secondary metabolites, including phenolics, flavonoids, saponins, terpenoids, and carotenoids, as reported in the literature [12].

Among these species, *Cochlospermum vitifolium* is notable as a medicinal tree, commonly referred to as algodão or pacotê. Native to South and Central America, *C. vitifolium* can attain heights of up to 5 m [12]. Multiple studies have documented its traditional use in managing hypertension, hepatitis, jaundice, hyperglycemia, and inflammatory disorders [13]. Furthermore, research has confirmed its efficacy in treating hepatitis, jaundice, and hyperglycemia [12, 14], as well as its anti‐inflammatory and analgesic properties [15], antihypertensive effects [14, 16], and hypoglycemic properties [17].

With regard to its application in the treatment of diseases and development of herbal medicines, it is always important to correlate the observed activities with the plant's metabolites and define some marker. The development of analytical techniques in metabolomic studies, particularly liquid chromatography coupled with high‐resolution mass spectrometry (LC‐HRMS), along with the expansion of available spectral libraries, has significantly accelerated the characterization of plant extracts. This progress facilitates the annotation of secondary metabolites, thereby reducing time and solvent use for isolating and screening for important biological activities [18].

Moreover, integrating these analytical techniques with bioinformatics tools, such as MzMine, MSDial, GNPS, and MetaboAnalyst, has greatly advanced data processing by automating peak detection and increasing the number of substances that can be annotated in each analysis. This integration enables researchers to correlate the chemical profiles of plant extracts with their corresponding biological activities [18, 19].

Although the uses of *C. vitifolium* and its reported biological activities are well documented in the literature, gaps remain in the chemical characterization of hydroethanolic extracts from its leaves and stem bark. Therefore, this study aimed to identify the substances present in the aforementioned extracts using a metabolomics approach and investigate antifungal, antioxidant, and cytotoxic properties.

## 2 Experimental Section

### 2.1 Plant Material

Leaves and stem bark of *C. vitifolium* were collected from a protected forest in the municipality of Assu, RN, Brazil (GPS: 5°35'00.5“S 36°56'40.0”W and 5°35'00.5“S 36°56'40.3”W) in July 2022. The plant materials were dried in a circulating air oven at 50°C, pulverized into powder and stored in a desiccator at room temperature until extraction. The voucher specimens were deposited in the herbarium of the Federal University of Rio Grande do Norte, registration number UFRN00028814. Botanist Carla Oliveira identified the species' parts. All necessary permissions for accessing and collecting genetic resources were obtained from SISBIO (83600) and SISGEN (A8772EF).

### 2.2 Extraction Procedure

Plant extracts 1% (w/v, 1 g of plant powder to 100 mL extract solvent) were prepared in ethanol:water (7:3, v/v), with ultrasonication for 30 min, followed by filtration, rotary evaporation, freeze‐drying, and storage at −20°C. For chemical profile analysis, a 5 mg sample of the prepared plant extract was dissolved in acetonitrile:water (7:3, v/v) and diluted to 1 mg/mL. Finally, the extract was filtered through a 0.22 µm PTFE membrane filter and transferred directly into a vial.

### 2.3 LC‐DAD‐ESI/MS‐MS Analysis

Elute Bruker UHPLC analyzed LC‐MS/MS analyses with a Kinetex XB‐C18 column (4.6 mm × 150 mm, 2.6 µm particle size) coupled to a diode array detector (DAD) and a compact sequential mass spectrometer with electron spray ionization (ESI‐QTOF‐MS/MS).

The mobile phase consisted of two solvents: acetonitrile (B) and water (A) both formic acid 0.1%, starting with 5% of phase B for 1 min, then increasing from 5% to 100% over 22 min, maintaining 100% for an additional 2 min, returning to the initial condition over 1 min, and finally maintaining that condition for 4 min. The flow rate was set at 0.8 mL/min, and the injection volume was 5 µL. During the first minute of each run, sodium formate (calibrant) was injected using an injection pump connected to the mass source.

The diode‐array detector was configured to detect wavelengths from 190 nm to 700 nm. The electrospray ionization (ESI) mass spectrometer was set with a capillary voltage of 4.5 kV, a source temperature of 220°C, and a drying gas flow of 9 L/min. It operated in both positive and negative ionization modes, with a mass detection range from *m*/*z* 50 to 1300 Da. Data acquisition and export were conducted using Data Analysis software version 6.1.

### 2.4 Data Analysis and Conversion

After completing the mass analysis, the raw data were converted to .mzML format using MSConvert from ProteoWizard [20]. The files were then imported into MSDial version 4.9 for data processing, following the parameters specified in Table S1. Subsequently, alignment results (spectra files) for each plant organ were exported in .MGF format and imported into Sirius software 6.2.2 for molecular annotations. The substances annotated were classified using the confidence levels established by Schymanski et al. (2014) [21]. These levels range from L1 to L5, with level L1 representing identification by a reference standard and level L5 indicating identification based solely on the exact mass. In this study, substances were assigned to confidence levels of L2 or L3. Level 2 denotes similarity with spectral libraries derived from fragmentation and is supported by existing literature, whereas level 3 is based on fragmentation data. The confidence levels assigned to these compounds are presented in Table S2.

### 2.5 GC‐MS Analysis

The chromatographic method was performed using an Agilent 8860 GC, coupled to an Agilent 5977B mass detector, and an HP‐5MS column (30 m × 0.25 mm × 0.25 µm). The samples were analyzed in splitless mode, with an injection volume of 1 µL. The chromatographic run method comprised the following temperature ramp: 0–5 min, 75°C; 5–41 min, 75°C–290°C; and 41–60 min, 290°C, totaling 60 min of analysis. The mass spectrometer used electron ionization at 70 eV, and an acquisition range of *m*/*z* 50–500.

For the identification of substances by GC‐MS, the alkane standard (C_10_–C_38_) was analyzed using the same method, and the retention index (Kovats index, KI) was calculated. The calculated indices were compared with indices of the same compounds identified by other articles in the literature and by NIST 17, which used the same methodology.

For derivatization, the method used was described by Vinod (2008) [22] with modifications. Then, 5 mg of dry extracts of *C. vitifolium* stem bark and leaves were weighed, 900 µL of acetonitrile was added, and the samples were placed in an ultrasonic bath for 60 min to solubilize them. The silylation solution (100 µL of BSTFA with 1% TCMS) was added to the solution, homogenized, and heated to boiling on a hot plate. Immediately thereafter, the vials were analyzed by GC‐MS.

### 2.6 Antioxidant Activity

ABTS and DPPH radical scavenging tests were used to determine the antioxidant activity of the hydroethanolic extracts, following guidelines with modifications [23]. For the assay, 40 µL of the hydroethanolic extract was added to a 96‐well microplate, then added 260 µL of ABTS solution. The reading was taken immediately at 734 nm using a microplate reader. The DPPH solution was prepared, and 200 µL was added to a microplate. Then, 40 µL of the test sample solution was added and incubated for 25 min, protected from light. After the elapsed time, the reading was taken at 515 nm. A Trolox standard curve was used, and the results were expressed as µmol Trolox/g extract. All experiments were performed in triplicate.

### 2.7 Total Phenolic Content (TPC)

The Folin–Ciocalteu method, with modifications [24], was used to determine the TPC of the leaves and stem bark extracts. This method added 25 µL of the sample and 75 µL of water to a 96‐well plate, followed by 25 µL of the Folin–Ciocalteau reagent (1:1 v/v). After 6 min, 100 µL of 7.5% w/v sodium carbonate (Na_2_CO_3_) was added, and the microplate was protected from light for 90 min. The absorbance was measured at 765 nm using a microplate reader (Epoch‐Biotek, Winooski, VT, USA). Gallic acid was used as the standard substance for quantification, and the TPC was expressed in µg GAE/g sample (micrograms of gallic acid equivalents per gram of sample).

### 2.8 Total Flavonoid Content (TFC)

The TFC in the extract was determined by a colorimetric method based on the reaction of flavonoids with aluminum chloride (AlCl_3_), as proposed by Vuong [25], with minor modifications. Fifty microliter of the extract was added to a 96‐well microplate, followed by the addition of 160 µL of ethanol, 20 µL of sodium acetate (C_2_H_3_NaO_2_, 8.2% w/v), and 20 µL of 1.8% (w/v) AlCl_3_. The sample was incubated in the dark for 40 min, and the absorbance was measured at a wavelength of 415 nm using a microplate reader. The standard used was quercetin, and the total flavonoid content was expressed as microgram of quercetin equivalents per gram of sample dry weight (µg QE/g sample).

### 2.9 Antifungal Activity

Antifungal activity was determined using the microbroth dilution assay according to the M27‐A2 guidelines from the CLSI. The crude hydroethanolic extracts (leaves/stem bark) were weighed in Eppendorf tubes and dissolved in DMSO to 25 mg/mL to obtain stock solutions. These were then diluted in RPMI 1640 medium to 1 mg/mL, maintaining the DMSO concentration below 5% (v/v). The RPMI 1640 medium (with phenol red, without sodium bicarbonate) was prepared by dissolving 10.4 g of dehydrated medium in 1 L of distilled water, adjusting the pH to 7.0 with 3‐[*N*‐morpholino]‐propanesulfonic acid (MOPS), and sterilizing the solution by vacuum filtration through a 0.22 µm membrane. To confirm sterility, aliquots were incubated at 37°C for 5 days before storage at 4°C. The fungal panel consisted of the clinical isolates *C. krusei*, *C. glabrata*, and *C. tropicalis*, and the reference strains *C. albicans* ATCC 10321 and *C. parapsilosis* ATCC 22019. All strains were cultured on potato dextrose agar (PDA) at 35°C for 48 h, and subcultures were prepared 48 h prior to testing. The inoculum was suspended in sterile 0.85% saline and adjusted to a 0.5 McFarland standard (approximately 10^6^ cells/mL), then diluted in RPMI to achieve a final concentration of 0.5 × 10^3^ to 2.5 × 10^3^ CFU/mL in each well.

Antifungal activity was assessed using the broth microdilution method in sterile 96‐well microplates. Each well in the first column received 100 µL of RPMI medium, and 100 µL of diluted extract or positive control, totaling 200 µL per well. Serial dilutions continued to the nineth column. The inoculum was added to all wells except the 10th column, which served as sterility control, while the 11th column contained only RPMI and the inoculum as the growth control. Plates were sealed with Parafilm M, incubated at 35°C for 48 h, after which fungal growth was visually evaluated. The minimum inhibitory concentration (MIC) was defined as the lowest concentration at which no visible fungal growth was observed.

Antifungal activity of the extracts was considered based on the Kuete classification [26] as: significant (MIC < 100 µg/mL), moderate (100< MIC ≤ 625 µg/mL) or weak (MIC > 625 µg/mL).

### 2.10 Cytotoxic Studies

The MTT assay was used to determine the cytotoxicity of the leaves and stem bark of *C. vitifolium*. L929‐CCL‐1 (murine fibroblasts from subcutaneous connective tissue) and MC3T3‐E1 preosteoblastic cells (obtained from American Type Culture Collection—ATTC, USA) were used for both samples.

The L929‐CCL‐1 cells were grown in Dulbecco's modified eagle's medium (DMEM) and α‐MEM for MC3T3‐E1, both containing 10% fetal bovine serum (FBS), 100 µg/mL of streptomycin, and 100 UI/mL of penicillin. Cells were incubated in a CO_2_ incubator at 37°C and 5% CO_2_ for 24 h. Next, the medium was removed, and a new medium containing FBS was added. These cells were treated with 10, 25, 50, and 100 µg/mL of the hydroethanolic extract for 24 h. Cells without DMSO and those treated with 1% DMSO served as control groups. Following the treatment, the cells were washed with PBS, and MTT reagent was added at a concentration of 12 mM. The cells were incubated for 4 h at 37°C with 5% CO_2_. Afterward, 100 µL of ethanol was added to each well containing MTT. Fifteen minutes later, the absorbance of the samples was measured at 570 nm using an ELISA reader.

### 2.11 Statistical Analysis

All experiments were conducted in triplicate. An alignment table was exported from MSdial and imported into MetaboAnalyst 6, the data were adjusted using Pareto scaling, and then a volcano plot, and principal component analysis (PCA) were constructed. Chord diagram was created in the R package. Minimum inhibitory concentration (MIC) values were visualized in GraphPad Prism 8, and statistically significant at *p* < 0.05.

## 3 Results and Discussions

### 3.1 Characterization of Chemical Constituents in the Hydroethanolic Leaves and Stem Bark Extracts of *C. vitifolium* by UHPLC‐MS/MS

In this study, 71 compounds were annotated from hydroethanolic leaves and stem bark extracts of *C. vitifolium* using the MSdial and Sirius software, with 44 from leaves and 27 from stem bark. These compounds included 41 flavonoids, 7 phenolic acids, 4 tannins, 2 terpenoids, 4 fatty acyl glycosides of mono‐ and disaccharides, 5 sugars, and 8 compounds belonging to other classes, including 1 purine, 1 amino acid, 2 saccharolipids, and *O*‐glycosyl compounds. Table 1 shows detailed information about the compounds for both organs, including peak number, retention time (minutes), protonated molecular mass (experimental and theoretical), molecular formulas, mass errors, fragment ions, and annotated compounds. This approach was used to separate and identify bioactive compounds from leaves and stem bark without the need for isolation or multiple purifications. All chromatograms, mass spectra, confidence levels, and references for all annotated substances are available in Table S2 and Figures S1–S71).

**TABLE 1:** Annotation of chemical components in the hydroethanolic leaves and stem bark extracts of *C. vitifolium* by UHPLC‐MS/MS in positive mode.

Leaves							
Number	RT (min)	[M + H]^+^	Mass theoretical	Error (ppm)	MS/MS	MF	Annotated
1	1.75	163.0602	163.0601	0.6	85.0281, 97.0278, 118.0862, 162.1126	C_6_H_10_O_5_	Glucosan
2	1.75	325.1140	325.1129	3.38	85.0283, 127.0385, 145.0496, 163.0605	C_12_H_20_O_10_	GlyTouCan: G99033OS
3	1.75	198.0970	198.0972	−1.00	127.0392, 145.0498, 163.0598	C_6_H_15_NO_6_	(2*R*,3*R*,4*R*,5*S*)‐5‐Amino‐6‐hydroperoxyhexane‐1,2,3,4‐tetrol
4	1.83	193.0711	193.0706	2.58	111.0441, 147.0657	C_7_H_12_O_6_	Quinic acid
5	1.95	136.0613	136.0617	−2.93	119.0356, 136.0620	C_5_H_5_N_5_	Adenin
6	2.11	254.1254	254.1234	7.87	85.0291, 145.0498, 153.0190, 197.0439	C_9_H_19_NO_7_	2‐*O*‐*D*‐Glucosaminyl glycerol
7	2.26	345.0831	345.0816	4.34	107.0125, 125.0235, 153.0187	C_14_H_16_O_10_	Theogallin
8	3.20	166.0857	166.0862	−3.01	103.0543, 120.0808	C_9_H_11_NO_2_	Phenylalanine
9	3.94	345.0830	345.0816	4.05	107.0127, 125.0234, 153.0189	C_14_H_16_O_10_	Theogallin
10	5.75	497.0941	497.0925	3.41	153.0188, 327.0734, 479.0872	C_21_H_20_O_14_	3,4‐di‐*O*‐Galloylquinic acid
11	6.14	433.1331	433.1340	−2.07	127.0384, 153.0190	C_18_H_24_O_12_	Licoagroside B
12	6.23	441.0998	441.1027	−6.57	127.0393, 153.0182, 315.0725	C_19_H_20_O_12_	[6‐(3,5‐Dihydroxyphenoxy)‐3,4,5‐trihydroxyoxan‐2‐yl]methyl 3,4,5‐trihydroxybenzoate
13	6.41	652.1155	652.1144 [M + H + H_2_O]^+^	1.68	277.0351, 465.0666	C_27_H_22_O_18_	Corilagin
14	6.54	595.1646	595.1656	−1.68	153.0206, 295.0607, 325.0721, 337.0720, 379.0833, 457.1132, 475.1205, 499.1157, 559.1401, 577.1572, 595.1655	C_27_H_30_O_15_	Violantin
15	6.79	455.1180	455.1184	−0.87	141.0543, 153.0150, 297.0623, 315.0730	C_20_H_22_O_12_	[6‐(2,4‐Dihydroxy‐6‐methylphenoxy)‐3,4,5‐trihydroxyoxan‐2‐yl]methyl 3,4,5‐trihydroxybenzoate
16	7.20	633.1041	633.1086	−7.10	153.0183, 319.0462	C_28_H_24_O_17_	Myricetin 3‐(6''‐galloylgalactoside)
17	7.30	449.1097	449.1078	4.23	299.0558, 329.0662, 353.0658, 395.0774, 413.0871, 431.0977	C_21_H_20_O_11_	Isoorientin
18	7.33	389.2188	389.2170	4.62	105.0337, 209.1543	C_19_H_32_O_8_	Lauroside E
19	7.49	481.1000	481.0976	4.98	153.0209, 319.0468	C_21_H_20_O_13_	Isomyricitrin
20	7.86	617.1146	617.1137	1.45	153.0190, 303.0516	C_28_H_24_O_16_	Quercetin 3‐(6''‐galloylglucoside)
21	7.99	433.1147	433.1129	4.15	283.0614, 313.0719, 367.0830, 397.0930, 415.1035	C_21_H_20_O_10_	Vitexin
22	8.17	465.1031	465.1028	0.64	303.0510	C_21_H_20_O_12_	Hyperin
23	8.60	601.1161	601.1188	−4.49	153.0182. 287.0556, 315.0721	C_28_H_24_O_15_	Kaempferol 3‐(6''‐galloylgalactoside)
24	8.60	435.0945	435.0922	5.74	303.0519	C_20_H_18_O_11_	Reinutrin
25	8.70	769.1230	769.1247	−2.21	153.0191 303.0528, 455.0621	C_35_H_28_O_20_	2‐{[2‐(3,4‐Dihydroxyphenyl)‐5,7‐dihydroxy‐4‐oxo‐4*H*‐chromen‐3‐yl]oxy}‐4,5‐dihydroxy‐6‐[(3,4,5‐trihydroxybenzoyloxy)methyl]oxan‐3‐yl 3,4,5‐trihydroxybenzoate
26	8.75	585.1247	585.1239	1.36	153.0193, 283.0618, 313.0729	C_28_H_24_O_14_	2‐[5,7‐Dihydroxy‐2‐(4‐hydroxyphenyl)‐4‐oxo‐4*H*‐chromen‐8‐yl]‐4,5‐dihydroxy‐6‐(hydroxymethyl)oxan‐3‐yl 3,4,5‐trihydroxybenzoate
27	8.85	449.1080	449.1078	0.44	303.0521	C_21_H_20_O_11_	Quercetin
28	8.85	303.0522	303.0499	7.58	153.0176, 303.0513	C_15_H_10_O_7_	Quercetin aglycone
29	8.93	373.2238	373.2221	4.55	109.1010, 135.1163, 193.1579, 211.1696	C_19_H_32_O_7_	Byzantionoside B
30	9.12	419.0965	419.0973	−1.90	287.0557	C_20_H_18_O_10_	Cyanidin 3‐arabinoside
31	9.29	525.2316	525.2330	−2.66	153.0184, 211.1696, 315.0714	C_26_H_36_O_11_	[3,4,5‐Trihydroxy‐6‐[4‐(2,6,6‐trimethyl‐4‐oxocyclohex‐2‐en‐1‐yl)butan‐2‐yloxy]oxan‐2‐yl]methyl 3,4,5‐trihydroxybenzoate
32	9.59	287.0569	287.0550	6.61	115.0545, 135.0453, 201.0551, 286.1468	C_15_H_10_O_6_	2'‐Hydroxygenistein
33	9.67	601.1201	601.1188	2.16	153.0192, 303.0519, 455.0624	C_28_H_24_O_15_	2‐{[2‐(3,4‐Dihydroxyphenyl)‐5,7‐dihydroxy‐4‐oxo‐4H‐chromen‐3‐yl]oxy}‐3,5‐dihydroxy‐6‐methyloxan‐4‐yl‐3,4,5‐trihydroxybenzoate
34	9.85	525.2348	525.2330	3.42	153.0189, 211.1699, 315.0710	C_26_H_36_O_11_	[3,4,5‐Trihydroxy‐6‐[4‐(2,6,6‐trimethyl‐4‐oxocyclohex‐2‐en‐1‐yl)butan‐2‐yloxy]oxan‐2‐yl]methyl 3,4,5‐trihydroxybenzoate
35	9.97	611.1358	611.1395	−6.05	147.0443, 303.0518	C_30_H_26_O_14_	Helichrysoside
36	10.06	641.1524	641.1501	3.58	145.0296, 177.0549, 303.0515	C_31_H_28_O_15_	Quercetin 3‐(6''‐ferulylglucoside)
37	10.28	595.1402	595.1446	−7.39	147.0441, 207.0654, 329.065	C_30_H_26_O_13_	(6‐{[2‐(3,4‐Dihydroxyphenyl)‐5‐hydroxy‐4‐oxo‐4*H*‐chromen‐7‐yl]oxy}‐3,4,5‐trihydroxyoxan‐2‐yl)methyl 3‐(4‐hydroxyphenyl)prop‐2‐enoate
38	10.46	625.1492	625.1552	−9.59	117.0338, 145.0287, 177.0551, 329.0683	C_31_H_28_O_14_	Luteolin 7‐(6''‐ferulylglucoside)
39	10.76	579.1454	579.1497	−7.42	147.0441, 313.0713, 579.1496	C_30_H_26_O_12_	Vitexin 2''‐*O*‐*p*‐coumarate
40	10.96	609.1632	609.1603	4.76	145.0281, 177.0554, 313.0718	C_31_H_28_O_13_	Vitexin 2''‐*O*‐(*E*)‐ferulate
41	12.67	333.0980	333.0969	3.30	137.0238, 137.0600, 167.0340, 209.0448, 259.0969, 287.0920, 315.0870, 333.0961	C_17_H_16_O_7_	2‐(3,4‐Dihydroxyphenyl)‐5‐hydroxy‐3,7‐dimethoxychroman‐4‐one
42	14.28	289.0840	289.0859	−6.57	115.0534, 135.0437, 287.1496	C_19_H_12_O_3_	4*H*‐Naphtho(1,2‐b)pyran‐4‐one, 6‐hydroxy‐2‐phenyl‐
43	15.51	453.3384	453.3363	4.63	407.3316, 453.3368	C_30_H_44_O_3_	3‐Oxooleana‐1,12‐diene‐28‐oic acid
44	15.51	506.3863	506.3840 [M + H + H_2_O]^+^	4.54	201.1645, 407.3335, 435.3299, 453.3392, 471.3497, 506.3863	C_30_H_48_O_5_	Arjunolic acid
Stem bark							
45	1.60	181.0710	181.0707	1.65	85.0277, 127.0385, 145.0501	C_6_H_12_O_6_	d‐Glucose
46	1.70	343.1230	343.1235	−1.45	85.0279, 109.0274, 127.0387, 145.0497, 163.0597, 181.0701	C_12_H_22_O_11_	Sucrose
47	1.75	163.0600	163.0601	−0.61	85.0271, 91.0389, 97.0277, 162.1135	C_6_H_10_O_5_	3‐(2,5‐Dioxabicyclo[2.1.0]pentan‐3‐yloxy)propane‐1,2‐diol
48	1.77	325.1140	325.1129	3.38	85.0278, 127.0388, 145.0493, 163.0604	C_12_H_20_O_10_	4‐*O*‐Beta‐d‐glucopyranosyl‐1,2‐anhydro‐d‐glucopyranose
49	1.85	193.0710	193.0707	1.55	111.0437, 147.0653	C_7_H_12_O_6_	Quinic acid
50	2.16	268.1040	268.1027	4.84	119.0347, 136.0615	C_9_H_17_NO_8_	(2*R*,3*R*,4*S*,5*S*,6*R*)‐6‐(Hydroxymethyl)‐3‐(3‐nitropropoxy)oxane‐2,4,5‐triol
51	2.70	171.0280	171.0288	−4.67	81.0344, 107.0123, 109.0270, 125.0246, 127.0408, 153.0140	C_7_H_6_O_5_	Gallic acid
52	4.80	199.0610	199.0601	4.52	128.9503, 140.0469, 158.0050	C_9_H_10_O_5_	Syringic acid
53	5.73	467.1190	467.1184	1.28	123.0442, 149.0224 153.0185, 213.0569, 231.0642, 259.0622, 287.0529, 305.0654	C_21_H_22_O_12_	Isoglucodistylin
54	6.13	291.0870	291.0863	2.40	123.0439, 139.0393, 147.0440, 207.0652	C_15_H_14_O_6_	Catechin
55	6.58	451.1220	451.1235	−3.32	107.0489, 149.0228, 153.0176, 215.0709, 243.0654, 271.0593, 272.0652, 289.0698, 290.0740	C_21_H_22_O_11_	Sinensin
56	7.04	467.1170	467.1184	−2.99	153.0186, 195.0283, 231.0657, 287.0557, 305.0663	C_21_H_22_O_12_	Isoglucodistylin
57	7.07	305.0670	305.0656	4.58	123.0439, 149.0231, 153.0179, 231.0654, 259.0608, 287.0562	C_15_H_12_O_7_	Taxifolin (dihydroquercetin)
58	7.20	459.0880	459.0922	−9.14	139.0389, 140.0424, 151.0382, 153.0175, 289.0703	C_22_H_18_O_11_	Epigallocatechin gallate
59	7.75	289.0710	289.0707	1.03	107.0489, 149.0233, 153.0183, 215.0705, 243.0649, 271.0594	C_15_H_12_O_6_	Dihydrokaempferol
60	8.17	465.1020	465.1028	−1.72	153.0169, 289.0690, 303.0494	C_21_H_20_O_12_	Isoquercetin
61	8.25	443.0940	443.0973	−7.44	123.0435	C_22_H_18_O_10_	Epicatechin gallate
62	8.55	305.0650	305.0656	−1.96	123.0439, 149.0237, 153.0191, 231.0659, 259.0602, 287.0548, 303.0159	C_15_H_12_O_7_	Taxifolin (dihydroquercetin)
63	8.88	435.1280	435.1286	−1.37	153.0178, 273.0757	C_21_H_22_O_10_	Prunin
64	9.08	463.0880	463.0871	1.94	317.0293	C_21_H_18_O_12_	3'‐Mono‐*O*‐methylellagic acid 4‐*O*‐alpha‐l‐rhamnopyranoside
65	9.40	587.1400	587.1395	0.85	153.0184, 273.0759, 315.0716	C_28_H_26_O_14_	Prunin 6''‐*O*‐gallate
66	9.60	433.1100	433.1129	−6.69	153.0189, 273.0757	C_21_H_20_O_10_	Cosmetin
67	9.65	289.0710	289.0707	1.03	107.0490, 153.0184, 215.0705, 243.0655, 271.0600	C_15_H_12_O_6_	Dihydrokaempferol
68	9.67	599.1140	599.1184	−7.34	177.0552, 311.0525	C_32_H_22_O_12_	8‐[5,7‐Dihydroxy‐2‐(4‐hydroxy‐3‐methoxyphenyl)‐4‐oxochromen‐8‐yl]‐5,7‐dihydroxy‐2‐(4‐hydroxy‐3‐methoxyphenyl)chromen‐4‐one
69	10.39	505.0970	505.0977	−1.38	171.0675, 285.0054, 317.0309	C_23_H_20_O_13_	6‐((2,7‐Dihydroxy‐8‐methoxy‐5,10‐dioxo‐5,10‐dihydrochromeno[5,4,3‐cde]chromen‐3‐yl)oxy)‐4,5‐dihydroxy‐2‐methyltetrahydro‐2H‐pyran‐3‐yl acetate
70	11.88	273.0760	273.0757	1.09	153.0183, 273.0763	C_15_H_12_O_5_	Naringenin aglycone
71	12.17	271.0610	271.0601	3.32	91.0546, 153.0191	C_15_H_10_O_5_	Apigenin aglycone

Abbreviations: MF, molecular formula; RT, retention time in minutes.

#### 3.1.1 Flavonoids

The LC–ESI–MS/MS analysis enabled the annotation of several flavonoids belonging to distinct subclasses based on a metabolomics approach, including *C*‐glycosides, *O*‐glycosides, and *O*‐acylated derivatives. Structural suggestions were based on characteristic MS^2^ fragmentation patterns, such as neutral losses of sugars, hydroxyl groups, and acyl moieties, which are diagnostic for these flavonoid types [27].

Flavonoids were the most numerous substances in both extracts, as can be seen in the chord diagram. In the leaves, it was possible to identify 16 *O*‐glycosides flavonoids (**16**, **19**, **20**, **22**, **23**, **24**, **25**, **28**, **30**, **33**, **35**, **36**, **37**, **38**, **40**, and **41**) and 5 *C*‐glycosides flavonoids (**14**, **17**, **21**, **26**, and **39**), while 5 *O*‐glycosides flavonoids (**55**, **56**, **60**, **63**, and **64**) and 3 *C*‐glycosides flavonoids (**53**, **65**, and **66**) were annotated in the stem bark.

Considering *C*‐glycosides flavonoids of leaves, peak **14**, annotated as violantin (*m*/*z* 595.1646 [M + H]^+^), MS^2^ spectrum revealed fragment ions at *m*/*z* 577.1572, 559.1401, 499.1157, and 475.1205, corresponding to successive losses of water (–18 Da) and sugar fragments (–120 Da) at *m*/*z* 379.0833. Isoorientin (**17**) and vitexin (**21**), both belonging to the *C*‐glycosyl flavone subclass, were also detected. Isoorientin showed a molecular ion at *m*/*z* 449.1097, with MS^2^ fragments at *m*/*z* 431.0977, 413.0871, and 395.0774, revealing successive losses of water. Vitexin, detected at *m*/*z* 433.1147, produced fragments at *m*/*z* 415.1035, 397.0930, and 367.0830, corresponding to dehydration and cross‐ring cleavages of the sugar moiety [28]. Compound **26** (*m*/*z* 585.1247 [M + H]^+^) was annotated as an 8‐*C*‐glycoside linked to a galloyl. Fragment produced was *m*/*z* 313.0729, 283.0618, and 153.0193, indicating neutral losses of a part of the sugar and a galloyl group. The vitexin 2''‐*O*‐*p*‐coumarate (**39**, *m*/*z* 579.1454 [M + H]^+^) showed ions at *m*/*z* 313.0713 resulting from the cleavage of the *p*‐coumaroyl portion and the sugar‐linked part (–266 Da).

The *O*‐glycosides flavonoids, myricetin 3‐(6''‐galloylgalactoside), were annotated (**16**, *m*/*z* 633.1041 [M + H]^+^) and produced a fragment at *m*/*z* 319.0462, associated with the loss of a sugar and galloyl moiety (–314 Da) [29]. Similar fragmentation patterns were observed for quercetin 3‐(6''‐galloylglucoside) (**20**, *m*/*z* 617.1146 [M + H]^+^) and kaempferol 3‐(6''‐galloylgalactoside) (**23**, *m*/*z* 601.1161 [M + H]^+^), which displayed aglycone ions at *m*/*z* 303.0516 and 287.0556, respectively.

Compound **35** (*m*/*z* 611.1358 [M + H]^+^) shows two fragment ions at *m*/*z* 303.0518 and 147.0443, corresponding to the quercetin aglycone and sugar cleavage. Isomyricitrin (**19**, *m*/*z* 481.1000 [M + H]^+^) and **22**, *m*/*z* 465.1031 [M + H]^+^) both exhibited characteristic aglycone fragments at *m*/*z* 319.0468 (myricetin) and 303.0510 (quercetin), following a neutral loss of 162 Da, typical of sugar‐linked.

Peak **24**, corresponding to *m*/*z* 435.0945 [M + H]^+^, displayed a fragment at *m*/*z* 303.0519, indicating cleavage of an *O*‐linked sugar and quercetin as the aglycone. Compound **25** (*m*/*z* 769.1230 [M + H]^+^) produced ions at *m*/*z* 455.0621, 303.0528, and 153.0191, attributed to successive losses of galloyl and glycosyl moieties. A similar fragmentation profile was observed for compound **33** (*m*/*z* 601.1201 [M + H]^+^), which yielded ions at *m*/*z* 455.0624 and 303.0519, corresponding to the *O*‐glycosidic bond and the galloyl substitution, respectively.

The quercetin aglycone (**28**, *m*/*z* 303.0522 [M + H]^+^) presented an intense fragment at *m*/*z* 153.0176, corresponding to a retro‐Diels–Alder (RDA) cleavage within the *C*‐ring, a hallmark of flavonol structures. Cyanidin 3‐arabinoside (**30**, *m*/*z* 419.0965 [M + H]^+^) generated a fragment at *m*/*z* 287.0557, reflecting the loss of an arabinose residue (−132 Da), typical of *O*‐glycoside anthocyanidins [30].

Peaks **36–38** and **40** corresponded to *O*‐acylated flavonoids bearing *p*‐coumaroyl or feruloyl substituents. Quercetin 3‐(6''‐ferulylglucoside) (**36**, *m*/*z* 641.1524 [M + H]^+^) releasing fragment ions at *m*/*z* 303.0515, consistent with the loss of glucose (−162 Da) and feruloyl (−176 Da) moieties. Peak **37** (*m*/*z* 595.1402 [M + H]^+^) displayed ions at *m*/*z* 329.065, 207.0654, and 147.0441, arising from losses of *p*‐coumaroyl and sugar residues. Compound **38** *(m*/*z* 625.1492 [M + H]^+^) showed fragments at *m*/*z* 329.0683, suggesting ester cleavage and release of the luteolin aglycone. Moreover, Peak **40** *(m*/*z* 609.1632 [M + H]^+^) exhibited ions at *m*/*z* 313.0718, corresponding to the presence of a feruloyl group and a sugar.

The flavonoids described here were annotated in the hydroethanolic stem bark extract of *C. vitifolium*. Isoquercetin (**60**), classified as an anthocyanidin‐3‐*O*‐glycoside, exhibited a protonated ion at *m*/*z* 465.1020. The MS^2^ spectrum shows fragment ions at *m*/*z* 303.0494, characteristic of loss of a hexose (162 Da), and presence of aglycone quercetin [31].

Catechin compounds were also detected, including epigallocatechin gallate (**58**) and epicatechin gallate (**61**), both belonging to the catechin gallate subclass. The fragmentation profiles were characterized by the presence of ions with galloyl cleavage [31]. Catechin (**54**) exhibits this chemical pattern, with retro‐Diels–Alder fragmentation [32].

Among the flavanones and flavanonols, naringenin (**70**) and dihydroquercetin (**57** and **62**) were annotated, together with dihydrokaempferol (**59** and **67**). All substances were reported in the literature for *C. vitifolium* [12]. Apigenin (**71**) showing fragment ions *m*/*z* 153, corresponds to RDA fragmentation in agreement with previously reported fragmentation pathways for this aglycone [33].

A galloyl glucoside, prunin 6''‐*O*‐gallate (**65**), precursor ion at *m*/*z* 587.1400. MS^2^ data revealed fragments at *m*/*z* 315.0716, 273.0759, and 153.0184, indicating stepwise cleavage of the galloyl and sugar substituents [34].

Isoglucodistylin (**53** and **56**), another *C*‐glycoside, protonated ion at *m*/*z* 467.119, and its fragmentation pattern included ions at *m*/*z* 305 and 287, whose MS^2^ spectrum cleavages within the sugar moiety, followed by the loss of water. Compound **64**, a flavonoid‐7‐*O*‐glucuronide, showed a parent ion at *m*/*z* 463.0880. The base peak at *m*/*z* 317.0293 reflected the loss of a rhamnose residue (146 Da). Sinensin (**55**) displayed an ion at *m*/*z* 451.122 with a fragmentation pattern comprising ions at *m*/*z* 289.0698 and 271.0593, attributed to consecutive losses of the sugar moiety (−162 Da) and water.

Finally, cosmetin (**66**), classified as an isoflavonoid *C*‐glycoside, presented an ion at *m*/*z* 433.1100 with fragment ions at *m*/*z* 273.0757 and 153.0189.

#### 3.1.2 Phenolic Acids

UHPLC‐MS/MS spectral analysis indicated the presence of seven phenolic acids, their glycosides, or other derivatives. Annotated peaks included quinic acid (**4**/**49**) for leaves and stem bark, respectively, as the main phenolic acids, alongside their glycosides such as 3,4‐di‐*O*‐galloylquinic acid (**10**), (**12**), (**15**), and the presence of tannins, such as theogallin (**7**), (**9**), (**13**). Quinic acid was annotated by protonated molecule [M + H]^+^ at *m*/*z* 193.0711, and its fragmentation *m*/*z* 147.0657, with a loss of 42 Da reference pattern CO_2_ [35].

Among phenolic acids and derivatives, peaks **10**, **12**, and **15** were annotated as galloylquinic acids, a gallotannin type presenting an ion [M + H]^+^ at *m*/*z* 497, 441, and 455, respectively, that released an MS^2^ fragment common at *m*/*z* 153 corresponding to gallic acid‐H_2_O, similar to tannins annotated peaks **7**, **9**, and **13**. Compounds **10** showed successive losses of two galloyl fractions, and 18 Da showed water loss based on MS^2^ *m*/*z* 479 and 153. Compounds **7**, **9**, **12, 13**, and **15** lost only galloyl moieties. These substances have been reported in the ethanol extract of *C. regium* leaves, corroborating the results obtained in this study [36] for stem bark, gallic acid (**51**), and syringic acid (**52**). The presence of phenolic acids, such as tannins and their glycosides, has been reported in other species of the genus *Cochlospermum* and in different organs [36, 37].

#### 3.1.3 Triterpenoids

Compound **43** showed the ion [M + H]+ at *m*/*z* 453.3363 and a fragment at *m*/*z* 407, corresponding to a neutral loss of HCOOH (46 Da), suggesting the molecular formula was C_30_H_44_O_3_ [38]. Peak **44** was annotated as arjunolic acid and showed the adduct [M + H + H_2_O]^+^, the theoretical mass of the compound is *m*/*z* 489. The fragmentation pathway 471 → 453 → 435 indicated the successive neutral losses of water (18 Da) [37, 39]. The compound was isolated from the rhizomes of *C. tinctorium* [40] and exhibited several biological activities [41].

#### 3.1.4 Fatty Acyl Glycosides

Peaks **18**, **29**, **31**, and **34** were annotated and classified as fatty acyl glycosides and disaccharides and appear on hydroethanolic leaves extract of *C. vitifolium*. These compounds are found in plants and are characterized by the bonding of a hydroxyl fatty acid or the carboxyl group of a fatty acid to a glycosyl portion. This class of substances includes articles on interactions between plants and the environment, such as plants and fungi and plants and insects, that affect plant defense [42].

### 3.2 GC‐MS Analysis

A total of 52 compounds were identified in different parts of *C. vitifolium*. These components were mainly classified into sugars, quinoline alkaloids, triterpenoids, phenolic acids, and fatty acids, identified in all parts of the study. Detailed results for the compounds, including retention time, Kovats index, % area, and molecular mass, are presented in Tables 2 and 3. Mass spectra and table containing the references are in Tables S3 and S4; Figures S72–S124. The majority of compounds found in the extracts were supporting material.

**TABLE 2:** Compounds identified in the hydroethanolic leaves extract of *C. vitifolium* by GC‐MS analysis.

Peak	RT	Compounds	Mass	% Area	KI_e_	KI_n_	KI_l_
1	12.038	Dihydroxyacetone, 2TMS	234.11	1.15	1216	NF	NF
2	12.503	Benzoic acid, TMS	194.07	40.45	1235	1242	NF
3	13.469	Glycerol, 3TMS	308.16	0.72	1273	1265	1279
4	14.798	Glyceric acid, 3TMS	322.14	0.33	1326	1318	1334
5	19.207	Pyrogallol, 3TMS	342.15	0.18	1527	1537	1535
6	19.896	2,3,4‐Trihydroxybutyric acid tetrakis(trimethylsilyl)	424.19	0.73	1562	NF	NF
7	20.893	d‐(−)‐Ribofuranose, tetrakis (trimethylsilyl) ether	438.21	0.08	1611	NF	1614
8	21.441	d‐(+)‐Ribono‐1,4‐lactone (*R*,*S*,*R*)‐, 3TMS	364.15	0.13	1641	NF	1639
9	24.005	d‐Allofuranose, pentakis(trimethylsilyl) ether	540.26	7.91	1781	NF	NF
10	24.29	Methyl α‐d‐glucofuranoside, 4TMS	482.23	2.97	1796	NF	1804
11	24.633	d‐(−)‐Fructofuranose, pentakis(trimethylsilyl) ether	540.26	3.87	1816	NF	1816
12	24.887	d‐(−)‐Tagatofuranose, pentakis(trimethylsilyl) ether	540.26	0.38	1832	NF	NF
13	25.026	β‐d‐(+)‐Talopyranose, 5TMS	540.26	3.22	1840	NF	NF
14	25.105	Methyl α‐d‐glucofuranoside, 4TMS	482.23	7.13	1845	NF	NF
15	25.19	d‐(+)‐Talofuranose, pentakis(trimethylsilyl) ether	540.26	3.32	1850	NF	1842
16	25.441	Quininic acid (5TMS)	552.26	10.36	1864	1863	1875
17	25.907	d‐Fructose, 5TMS	540.26	1.49	1891	NF	1881
18	26.09	α‐d‐(+)‐Talopyranose, 5TMS	540.26	4.20	1902	NF	NF
19	26.816	Gallic acid, 4TMS	458.17	9.80	1947	1962	1954
20	27.431	β‐d‐Allopyranose, 5TMS	540.26	0.78	1985	NF	NF
21	27.822	Palmitic acid, TMS	328.27	0.36	2009	2015	NF
22	30.742	Stearic acid, TMS	356.31	0.08	2202	2207	NF
23	44.548	Stigmast‐5‐ene, 3β‐(trimethylsiloxy)‐, (24*S*)‐	486.42	0.36	3288	NF	NF

Abbreviations: KIe, Kovats Index experimental; KI_l_, Kovats Index literature; KIn, Kovats Index NIST; NF, not found; RT, retention time in minutes.

**TABLE 3:** Compounds identified in the hydroethanolic stem bark extract of *C. vitifolium* by GC‐MS analysis.

Peak	RT	Compounds	Mass	% Area	KI_e_	KI_n_	KI_l_
1	12.496	Benzoic acid, TMS	194.07	32.61	1235	1242	NF
2	13.472	Glycerol, 3TMS	308.16	3.37	1273	1265	1279
3	14.796	Glyceric acid, 3TMS	322.14	0.20	1326	1318	1334
4	15.608	Erythrono‐1,4‐lactone, (*E*)‐, 2TMS	262.10	0.11	1362	NF	NF
5	18.251	Malic acid, 3TMS	350.14	0.18	1481	1479	1481
6	18.823	l‐Threitol 4TMS	410.21	0.11	1507	1491	1509
7	19.897	2,3,4‐Trihydroxybutyric acid tetrakis(trimethylsilyl)	424.19	0.93	1562	NF	NF
8	21.441	d‐(+)‐Ribono‐1,4‐lactone (*R*,*S*,*R*)‐, 3TMS	364.15	0.11	1641	NF	1639
9	22.044	Arabinofuranose, 1,2,3,5‐tetrakis‐*O*‐(trimethylsilyl)	438.21	0.11	1673	NF	NF
10	23.999	d‐Allofuranose, pentakis (trimethylsilyl) ether	540.26	4.45	1780	NF	NF
11	24.284	Methyl α‐d‐glucofuranoside, 4TMS	482.23	2.88	1796	NF	1804
12	24.638	d‐(−)‐Fructofuranose, pentakis (trimethylsilyl) ether	540.26	5.97	1817	NF	1816
13	24.801	d‐(+)‐Talofuranose, pentakis (trimethylsilyl) ether	540.26	0.36	1826	1822	1828
14	24.881	d‐(−)‐Ribofuranose, tetrakis (trimethylsilyl) ether	438.21	0.39	1831	NF	1826
15	25.03	β‐d‐(+)‐Talopyranose, 5TMS	540.26	3.04	1840	NF	1852
16	25.465	Quininic acid (5TMS)	552.26	14.61	1866	1863	1875
17	25.754	l‐Glucono‐1,4 lactone (2*S*,3*R*,4*S*,5*S*)‐,4TMS	466.20	0.98	1882	NF	NF
18	25.947	*β*‐*D*‐(+)‐Mannopyranose, 5TMS	540.26	2.17	1893	NF	1888
19	26.096	*α*‐*D*‐Allopyranose, 5TMS	540.26	4.33	1902	NF	NF
20	26.145	*D*‐Altrose, 5TMS	540.26	1.43	1905	1911	NF
21	26.779	Gallic acid, 4TMS	458.17	1.51	1945	NF	1954
22	27.432	β‐d‐Allopyranose, 5TMS	540.26	1.14	1985	NF	NF
23	27.752	d‐Gluconic acid, 6TMS	628.29	0.39	2005	1997	NF
24	27.823	Palmitic acid, TMS	328.27	0.21	2009	2015	NF
25	35.346	Sucrose, 8TMS	918.43	11.94	2543	NF	2543
26	36.388	d‐(+)‐Turanose, octakis (trimethylsilyl) ether	918.43	0.41	2626	NF	NF
27	36.723	Lactose, 8TMS	918.43	2.89	2653	NF	NF
28	38.379	3‐α‐Mannobiose, octakis (trimethylsilyl) ether	918.43	0.92	2792	NF	NF
29	38.798	d‐Lactose, 8TMS	918.4	0.51	2824	NF	NF
30	40.81	Galactinol, nonakis (trimethylsilyl) ether	194.07	0.46	3008	2946	NF

Abbreviations: KIe, Kovats Index experimental; KI_l_, Kovats Index literature; KIn, Kovats Index NIST; NF, not found; RT, retention time in minutes.

GC–MS analysis of *C. vitifolium* identified benzoic acid as the most abundant compound in both leaves and stem bark, accounting for 40.45% and 32.61% of the extracts, respectively. In the leaves, other major constituents included quininic acid (10.36%), gallic acid (9.80%), d‐allofuranose (7.91%), and methyl α‐d‐glucofuranoside (7.13%). The stem bark was characterized by high levels of quininic acid (14.61%), sucrose (11.94%), and fructofuranose (5.97%). While benzoic acid is a phenolic compound commonly found in various plant species, no previous reports have documented its identification or annotation in other *Cochlospermum* species, even when the search was extended to related genera within the Bixaceae family, such as *Bixa orellana* [43]. Benzoic acid is also recognized as a constituent of other plant‐derived substances [44, 45]. Gallic acid was observed by both chromatographic techniques in the present study. Gallic acid was detected in this study using both chromatographic techniques and is frequently observed in species of the genus and across different plant tissues. Previous studies have reported that gallic acid exhibits antioxidant activity, contributing to hypoglycemic and genoprotective effects [46], as well as antibacterial [47] and antifungal [48] properties.

Alkaloids are reported in other species of the genus [49]. Quininic acid, a quinoline alkaloid, has been identified in the leaves and stem bark of *C. vitifolium*. The fatty acids palmitic and stearic acids were identified in the hydroethanolic leaf extract, whereas only palmitic acid was detected in the stem bark extract. Interestingly, this compound was described to have hypoglycemic activities [46].

However, based on the identification, it is possible to observe the presence of various monosaccharides with different abundances in the parts of the plant studied. Indeed, a sugar profile was reported in a study conducted by Vinod et al. on the presence of neutral sugars and uronic acids in gum kondagogu, an exudate extracted from the species *Cochlospermum gossypium* rich in polysaccharides and important to plant defense [22, 50, 51]. In another study using crude extracts and polysaccharide fractions, these compounds have been reported in the roots of *Cochlospermum tinctorium*, with various percentages of monosaccharides [52]. The methodology used in the author's work was similar to that of the present study. The present study observed a significantly lower abundance of sugars. This suggests that genetic variability between species and the type of plant part used may explain the discrepancy.

### 3.3 Statistical Analysis

**FIGURE 1:**
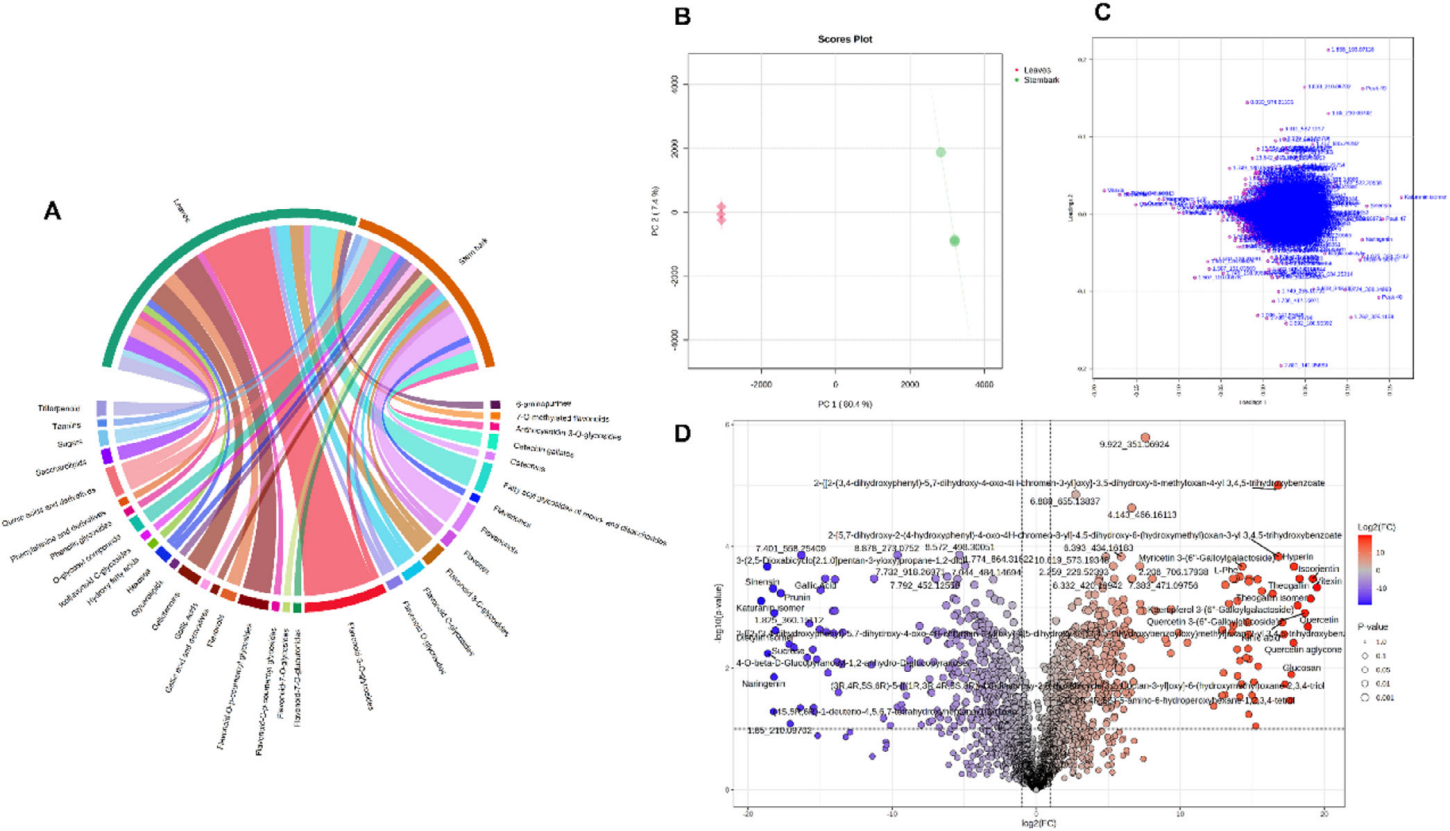
Chord diagram, PCA, loading plot and volcano plots of leaves versus stem bark of *C. vitifolium* by UHPLC‐MS/MS. Using log2 and a *p*‐value of 0.001, the upregulated and downregulated metabolites are shown in red and blue, respectively. The red circles indicate compounds that are significantly higher in leaves than in stem bark.

Chord diagram illustrates the presence of compound classes annotated by UHPLC‐MS/MS (Figure 1A); principal component analysis (PCA) was performed to assess the variability in metabolites between leaf and stem bark extracts. The PCA plot shows a clear separation among the tissues, 80.4% and 7.2% of the variation were explained by PC1 and PC2, respectively (Figure 1B). As shown in the loading graph (Figure 1C), separation is attributed to the presence of certain annotated metabolites. Chemical profile analysis revealed the occurrence of flavonoid glycosides, such as vitexin and isovitexin, and the tannin theogallin in the leaves. For stem bark, flavonoid aglycones, such as dihydroquercetin (**57**/**62**), naringenin (**70**), apigenin (**71**), and dihydrokaempferol (**59**/**67**) were present. This indicates that the metabolic constituents and their distribution in the leaves and stem bark of *C. vitifolium* varied significantly.

Differential analysis using volcano plots in Figure 1D shows differences in metabolites annotated for leaves (red) versus stem bark (blue). A circle represents each metabolite in the samples. Starting from zero on the *X*‐axis, the volcano plot is divided into left and right sides, which are represented by the colors blue and red, respectively. The statistically significant compounds are highlighted (above the dotted line) in red and blue, the compounds annotated as significant for a given plant organ.

### 3.4 Antifungal Activity

**FIGURE 2:**
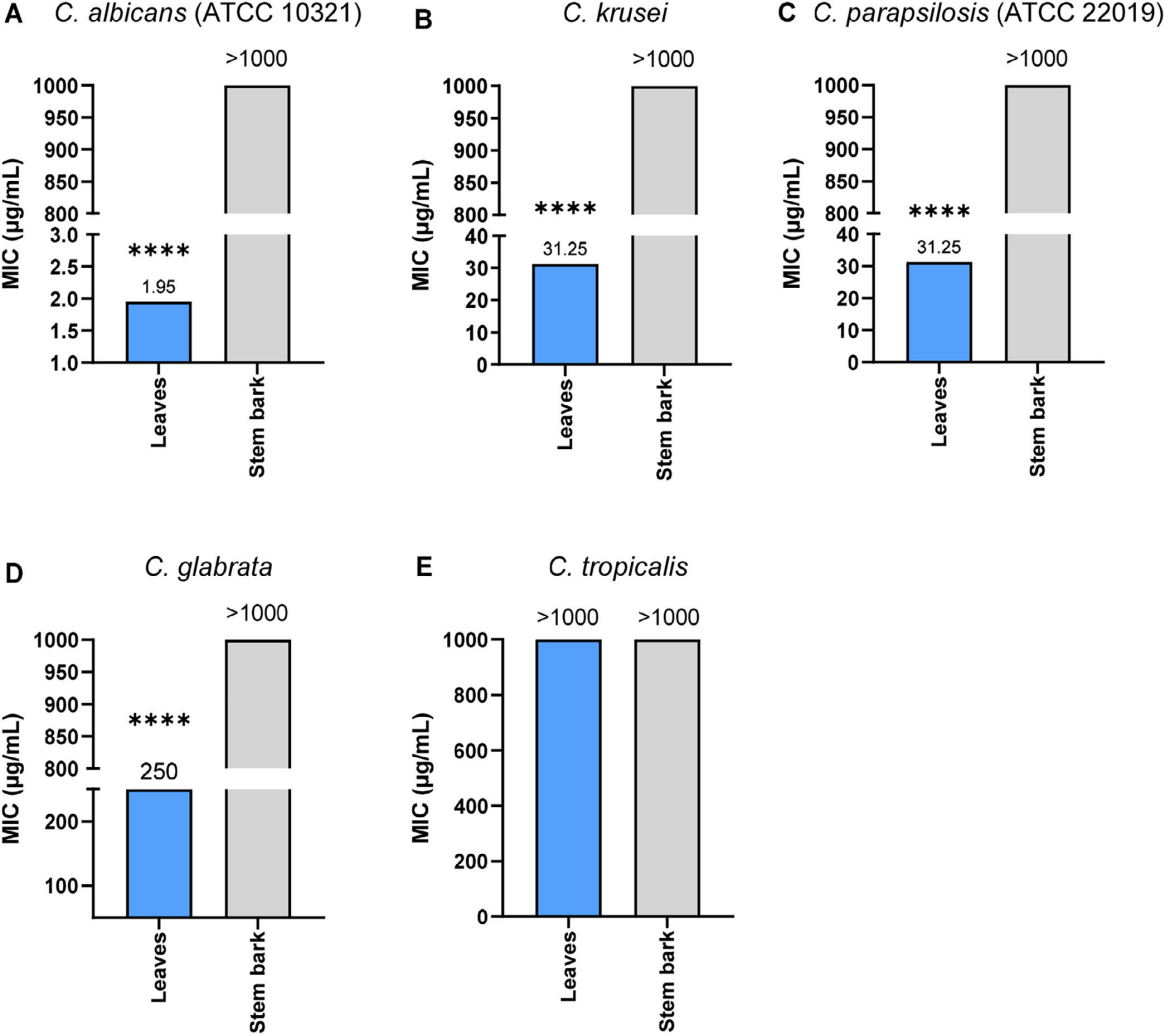
MIC values (µg/mL) of hydroethanolic extracts of *C. vitifolium* for leaves (blue) and stem bark (gray). (A) *C. albicans*; (B) *C. krusei*; (C) *C. parapsilosis*; (D) *C. glabrata*, and (E) *C. tropicalis*. Statistical differences between plant parts are represented by asterisks. *T*‐test independent correction significantly different four asterisks ^****^ *p* < 0.0001. Data are presented as means of triplicates.

Hydroethanolic extracts of *C. vitifolium* were evaluated against clinically relevant *Candida* spp. [9, 53, 54]. The extracts inhibited all tested strains, with MICs ranging from 1.95 µg/mL to 1000 µg/mL (Figure 2). The leaf extract demonstrated strong activity against *C. albicans* ATCC 10321 (MIC 1.95 µg/mL), whereas *C. glabrata* required a higher concentration for inhibition (MIC 250 µg/mL), likely reflecting its status as a clinical isolate with greater intrinsic resistance. The lack of inhibition against *C. tropicalis* (MIC > 1000 µg/mL) highlights the interspecific variability in susceptibility to plant‐derived metabolites. The identical MIC values (31.25 µg/mL) observed for *C. krusei* (clinical isolate) and *C. parapsilosis* (ATCC 22019) suggest that the active compounds may operate through mechanisms independent of common antifungal resistance, aligning with established resistance patterns of *Candida* species to standard antifungal agents [55, 56].

In contrast, the stem bark extract exhibited MIC values greater than 1000 µg/mL against all *Candida* strains tested. Variations in antifungal activity are generally associated with factors such as plant collection site, extraction solvent, plant species, and plant part utilized [57]. In this study, the greater inhibitory effect of the leaf extract compared to the stem bark extract is attributable to differences in chemical composition, particularly the higher phenolic and flavonoid content in the leaf extract, as previously discussed.

Climatic and environmental factors significantly influence plant metabolism and, consequently, antifungal activity. A study by Inácio et al. analyzing *C. regium* root extracts found that antimicrobial activity increases during fall and winter, attributed to water stress experienced by the species in these seasons. The study also noted that less fertile soils and mature plants enhance activity [58]. These findings corroborate the present results, as the plant material was collected at the end of the rainy season in the Caatinga geographical domain.

The antifungal activity of *C. vitifolium* extracts can be explained by the components annotated and identified by chromatographic techniques. The chemical profile detected by mass spectrometry analysis was highly significant for identifying antifungal activity. The weaker activity of the stem bark extract compared to the leaf extract can be attributed to subinhibitory concentrations of essential chemicals [48]. Although several compounds are shared between both parts of the plant, their relative abundances differ. This can be explained by dilution due to the higher sugar content in the bark, as shown by the area percentages of the organs in the GC analysis. This factor likely reduces the concentration of active compounds, thereby affecting their ability to reach the MIC required to suppress *Candida* growth. In addition, fungi such as *Candida* can increase their virulence, metabolic flexibility, and resistance to antifungals in media containing higher nutrient levels [59].

In addition to common compounds such as gallic, benzoic, and palmitic acids, which were more abundant in the leaves, chromatographic analyses identified unique compounds in the leaf extract, including pyrogallol, stigmast‐5‐ene, and stearic acid (GC‐MS), as well as corilagin and myricetin (LC‐MS/MS). These unique constituents may contribute to the higher antifungal activity observed. Gallic acid and its derivatives, such as hydrolyzable tannins, have demonstrated antifungal potential against *C. krusei*, *C. glabrata*, *C. tropicalis*, and *C. albicans* in LC‐MS/MS and GC‐MS analyses of *C. regium* roots [48]. Notably, these substances are also present in the hydroethanolic extracts of *C. vitifolium* leaves and stem bark.

Gallic acid exhibited MICs below 100 µg/mL against *C. glabrata*, *C. albicans*, and *C. tropicalis*, with the lowest value of 12.5 µg/mL for *C. albicans*, comparable to fluconazole at the same concentration. Additionally, at 50 µg/mL, gallic acid inhibited CYP51 and squalene epoxidase, enzymes involved in ergosterol biosynthesis, resulting in fungal membrane degradation similar to that observed with fluconazole at 12.5 µg/mL [60]. Benzoic acid demonstrated MIC values of 62.5–250 µg/mL against *Magnaporthe oryzae*, *Rhizoctonia solani*, and *Sclerotinia sclerotiorum* [61].

Pyrogallol was tested against *C. glabrata* and showed an MIC of 32 µg/mL for most reference strains and clinical isolates. It also demonstrated synergy with fluconazole, reducing its MIC by up to 128‐fold. The rationale for this potentiation of the azole lies in genetic tests and rhodamine‐based assays, which showed that pyrogallol inhibits the efflux pump that expels fluconazole from the fungus by targeting the genes that encode it (CgCDR1, CgCDR2, and CgPDR1) [62].

Although no specific trials for stigmast‐5‐ene were identified, a South African study evaluated phytosterols, including stigmasterol, against *Aspergillus flavus*, *Penicillium digitatum*, and *Fusarium verticilloides*, reporting MICs of 14–18 µg/mL, which are comparable to those of amphotericin B (13–14 µg/mL). Given the chemical similarity between stigmast‐5‐ene and stigmasterol, it is plausible that stigmast‐5‐ene contributed to the enhanced antifungal activity observed in the leaf extract [63].

Corilagin, a hydrolyzable tannin, demonstrated an MIC equivalent to that of amphotericin B for two *C. glabrata* strains and 500 µg/mL for *C. albicans* in a 2014 study [64]. The flavonol myricetin inhibited biofilm formation by 50% in three *C. albicans* strains, including a reference strain and two clinical isolates, and reduced the expression of genes regulating hyphal growth (RAS1, CYP1, and EFG1). In vivo, myricetin decreased fungal load and increased survival rates in larvae infected with *C. albicans* [65].

Beyond myricetin, numerous flavonoids have been reported to exhibit antifungal activity in the literature. In this study, both leaf and stem bark extracts contained glycosylated flavonoids at O and C positions, a common feature in plants [66]. Compounds annotated exclusively in the leaves, such as kaempferol 3‐(6''‐galloylgalactoside), quercetin 3‐(6''‐galloylglucoside), vitexin, and isoorientin, may contribute to the observed differences in antifungal activity compared to the stem bark. These compound classes are active against *Candida* spp. [67]. The ethyl acetate fraction of *C. regium* roots contained four glycosylated kaempferols with anti‐*Candida* activity (500–1000 µg/mL) [48], as well as catechins [68] and triterpenoids [69], which may exert fungicidal and fungistatic effects against various *Candida* strains.

Within the flavonoid spectrum, catechins such as epigallocatechin gallate (EGCG), epicatechin gallate, and catechin were detected exclusively in the stem bark extract. EGCG has been evaluated against *Candida* and *Trichophyton* species, with studies elucidating its antifungal mechanisms, including disruption of osmotic integrity, inhibition of dihydrofolate reductase, suppression of hyphal growth, and synergism with conventional azoles [70]. Arjunolic acid, a triterpene saponin identified only in the leaf extract, exhibited MIC values of 50 µg/mL against *C. krusei*, *C. parapsilosis*, and *C. albicans* [71]. No antifungal activity data was available for 3‐oxoolean‐1,12‐diene‐28‐oic acid.

No previous reports of antifungal activity have been reported for the plant species examined in this study. Comparative analysis of anti‐*Candida* activity revealed that the leaf extract of *C. vitifolium* exhibited lower MIC values than the stem bark, indicating greater anti‐fungal potency. A similar study with ethanolic extracts of *C. regium* leaves reported MICs of 64 µg/mL for *C. krusei*, 1,024 µg/mL for *C. glabrata* [72], and 500 µg/mL for *C. tropicalis* [73]. Carvalho et al. [48] demonstrated the superior antifungal activity of fractionated extracts and isolated compounds from *C. regium*. Although the crude extract of *C. vitifolium* yielded promising results, fractionation was not performed, precluding further investigation of molecular interactions, mechanisms of action, and the potential for synergistic effects on microbial cell membrane permeability [74].

### 3.5 Antioxidant Activity Analysis

The total phenolic and flavonoid content were determined using conventional methodologies described in the methodology section. The total phenol content and total flavonoid content results for *C. vitifolium* are shown in Table 4 below. The hydroethanolic extract of the leaves showed a TPC of 371 µg GAE/g and 328 µg QE/g for flavonoids. The value obtained from the stem bark was approximately 2.5 times lower for TPC and 6 times lower for flavonoid content compared to the leaves. In the study by Sarmento‐Filha (2022) [37], *C. vitifolium* flowers were extracted with solvents of varying polarity, yielding TPC values ranging from 35.17 mg to 525.90 mg GAE/g.

**TABLE 4:** Total phenolic content (TPC) (microgram equivalent of gallic acid/g) and total flavonoid content (TFC) (microgram equivalent of quercetin/g) of leaves and stem bark.

	Total content		Scavenging radicals	
Plant part	Phenolics (µg GAE/g)	Flavonoids (µg QE/g)	DPPH (mmol Trolox/L)	ABTS (mmol Trolox/L)
Leaves	366 ± 5.32	328 ± 9.32	113 ± 0.04	0.240 ± 0.00106
Stem bark	132 ± 3.39	55 ± 3.52	—	0.234 ± 0.00171

*Note*: DPPH and ABTS scavenging activity of extracts of *C. vitifolium*. Data are presented as mean ± standard deviation.

The aerial parts of the *C. planchonii* species showed TPC and TFC values of 202.64 mg GAE/g and 47.20 ± 0.71 mg RE/g, respectively, using ultrasound extraction [75]. The roots of *C. angolense* were obtained between 208.11 and 374.11 mg/g of phenolics extracted with a mixture of ethanol and water (1:1) by decoction and pressurized liquid extraction [76].

As there are no records in the literature on the leaves and stem bark of *C. vitifolium*, there is a discrepancy in the phenolic and flavonoid content between our results and other studies. This disparity can be attributed to differences in plant parts, solvents, and extraction techniques [77]. Variations in TPC and TFC levels between plants can also be caused by differences in soil composition, nutritional stress, as well as biotic and abiotic factors, such as feeding by seasonal insects or herbivorous animals, interaction with seasonal pathogens and diseases, and the availability of light, water, and temperature [78]. All these factors can also influence other metabolite levels and, consequently, the chemical composition of plants. Other factors that can contribute to differences in phenolic content include climatic and geographical variables, species‐specific characteristics, plant ripeness, and storage time [79]. In addition, increases or decreases in phenolic compounds directly affect the plant's antioxidant activity, as many phenolics contribute to it [80]. About the collected samples, fungal infection and herbivory were clearly noted in some leaves.

The DPPH and ABTS activity of the extracts obtained from the leaves and stem bark was assessed. The values were calculated using the standard curve of Trolox. Table 4 summarizes the values obtained. The leaf extract showed scavenging activity, with DPPH and ABTS values of 113 mmol Trolox/L and 0.240 mmol Trolox/L, respectively.

The literature reports published studies using DPPH assays and other antioxidant evaluation methods, such as iron‐chelating activity, copper‐chelating activity, superoxide radical scavenging activity, and hydroxyl radical scavenging activity, for the flowers of the species [37]. There are no reports in the literature on the antioxidant activity of the leaves of *C. vitifolium*.

A recent study evaluated the antioxidant potential of the barks and heartwood of *C. vitifolium*, demonstrating 80% inhibition of the DPPH radical at 6.25 mg/mL for the barks and 3.125 mg/mL for the heartwood [46]. These results highlight the antioxidant potential of this species' bark, though they cannot be directly compared with the current study due to the absence of Trolox curve.

Further expanding the research on the species, the authors demonstrated that the extraction of *Cochlospermum angolense* roots using pressurized liquid extraction (PLE) with 50% ethanol at 50°C–200°C showed greater DPPH radical scavenging activity than other extraction solvents. The values obtained ranged from 1284 µmol to 1488 µmol Trolox/g extract. Likewise, ABTS also presented high values, ranging from 3387 µmol to 4979 µmol Trolox/g extract. Compared to our results, the PLE technique provides better extraction than conventional techniques such as decoction [76]. The leaves of *C. planchonii* inhibited the radical ABTS 43.00 µmol Trolox E/L [81].

The antioxidative activity of different extracts from the leaves and rhizomes of *C. planchonii* and *C. tinctorium* was also tested, obtained by ultrasound‐assisted extraction and decoction, which showed antioxidant activity against the DPPH radical scavenger in the range of 10–124 mg Trolox equivalents/g [11]. In addition, the aqueous crude extract of *C. tinctorium* roots shows dose‐dependent DPPH radical scavenging [52]. However, upon purification to obtain fractions rich in polysaccharides, the radical‐scavenging activity is significantly reduced.

Although both extracts contain flavonoids and phenolics in their chemical composition, as observed in the compounds present in the LC‐MS/MS and GC‐MS analyses, such as gallic acid and others, which may contribute to their antioxidant activity [46], the hydroethanolic extract from the leaves obtained higher inhibition of DPPH and ABTS.

### 3.6 Cytotoxicity

**FIGURE 3:**
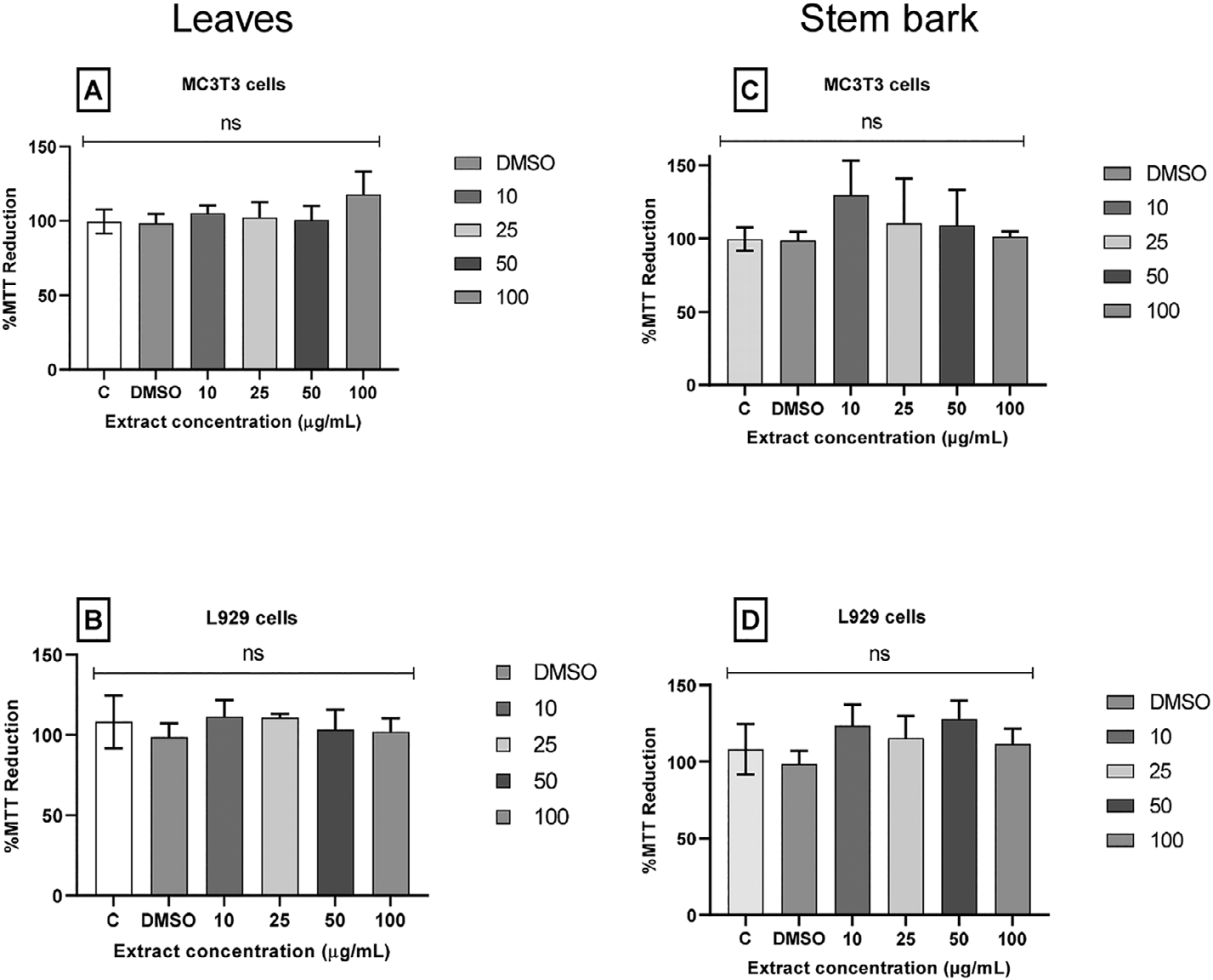
The hydroethanolic extract from the organs of species *C. vitifolium* was evaluated for cytotoxicity in MC3T3 and L929 cells, at concentrations 10, 25, 50, and 100 µg/mL. To leaves, (A) MC3T3 cells, (B) L929 cells, and stem bark, (C) MC3T3 cells, and (D) L929 cells. Results are presented as means ± SD; one‐way ANOVA, *n* = 3; when *p* < 0.05 versus negative control; Dunnett's test. ns = not significant.

An MTT assay was performed to evaluate the hydroethanolic extracts from the bark and leaves of *C. vitifolium*. Figure 3A–D demonstrates that neither extract‐induced cytotoxicity in either MC3T3 pre‐osteoblasts or L929 fibroblasts at all tested concentrations (10–100 µg/mL). The lack of cytotoxicity is supported by cell viability rates above 80% [82]. Dimethyl sulfoxide (DMSO), the solvent used for extract solubilization, was included as a control and did not induce cytotoxicity in the tested cells. In vitro cytotoxicity assays are critical for validating the biological safety of bioactive compounds and for confirming that therapeutic concentrations do not compromise host cell integrity. The L929 and MC3T3‐E1 cell lines were selected for their established sensitivity and stability in toxicological screening [83]. The results demonstrate that *C. vitifolium* extracts maintain high cellular viability at all tested concentrations, supporting a favorable biocompatibility profile. The absence of cytotoxicity is particularly significant when considered alongside the observed antifungal activity against *Candida* spp. and the antioxidant properties of the extracts, especially those derived from the leaves.

Currently, no published reports of cytotoxicity assays involving *C. vitifolium* bark or leaf extracts are available. Consequently, comparative analysis relies on data from studies of plant extracts within the same genus. The present findings are consistent with those of Leme et al. (2017), who reported no toxicity of *C. regium* leaf extracts in VERO cells at concentrations up to 150 µg/mL [73]. In contrast, the aqueous extract of *C. regium* roots exhibited dose‐dependent cytotoxicity in CHO‐K1 cells (EC_50_ = 1.5 mg/mL), inducing apoptosis and reducing cell proliferation [84]. Notably, this concentration is approximately 10 times higher than that employed in the current study.

In contrast, studies conducted on plants of the same genus indicated that extracts from *Cochlospermum* plants are cytotoxic to tumor cells. Previous studies have shown that ethanolic extracts from *C. regium* leaves inhibit the growth of HeLa cells [73], while gold nanoparticles synthesized from *C. gossypium* gum exhibit cytotoxicity against B16F10 melanoma cells [85]. These results demonstrate the antitumor potential of components present in the *Cochlospermum* genus and open up another field of research for *C. vitifolium* extracts.

Findings from this study, together with research on other *Cochlospermum* species, indicate that extract cytotoxicity is influenced by multiple factors, including the species and plant part utilized, which affect the metabolite composition, such as flavonoids, tannins, and saponins. Additionally, extract concentration significantly impacts cytotoxicity. These observations further support the efficacy and safety studies of using these extracts in biological systems.

## 4 Conclusions

In summary, this study is the first to simultaneously determine the complete chemical profiling, cytotoxicity, and antifungal activities of different *C. vitifolium* organs. Antioxidant assays showed higher activity for the leaves than in the stem bark extract. The results demonstrate that the investigated organs, particularly the leaves, exhibit significant antifungal properties, as evidenced by MIC assays against *C. krusei*, *C. glabrata*, *C. albicans*, and *C. parapsilosis*. The leaf extract inhibited the growth of *C. albicans* at 1.95 µg/mL, indicating strong antifungal activity. These findings suggest that this extract has potential as a future antifungal alternative, especially given the resistance of these strains to current drugs such as azoles. Further research should include testing fractions or isolated compounds and comparing their activity with the crude extract, as well as conducting in vivo experiments with the most promising candidates to reach more definitive conclusions regarding safety and antifungal efficacy.

## Author Contributions

**Francisco A. S. D. Pinheiro**: writing – original draft, methodology, investigation, statistical analysis. **Addison R. Almeida**: writing – review and editing, validation, formal analysis. **Matheus S. Menezes**: methodology, review and editing. **Gislene Ganade**: review and editing, resources. **Thomaz Rocha**: methodology, review and editing. **Weslley S. Paiva**: methodology, review and editing. **Hugo A. O. Rocha**: methodology, review and editing. **Cristiane F. de Assis**: methodology, review and editing. **Laila S. Espindola**: writing – review and editing, resources. **Leandro S. Ferreira**: review and editing, supervision, project administration, investigation, resources.

## Conflicts of Interest

The authors declare no conflicts of interest.

## Supporting information




**Supporting File 1**: cbdv71217‐sup‐0001‐SuppMat.docx. All data generated and analyzed during this study are included in this article and Supporting Information. Further details about this related data are available upon request to the corresponding author.

## Data Availability

The data that support the findings of this study are available from the corresponding author upon reasonable request.
