# Supplementary material for: Investigating the Chemical, Antifungal, and Cytotoxic Properties of Cochlospermum vitifolium Leaves and Stem Bark Extracts
Source: Chem Biodivers. 2026 Apr 24;23:e03856. doi: 10.1002/cbdv.202503856 (PMC13108566; doi:10.1002/cbdv.202503856)
Supplement: Supplementary file 1 — Supporting File 1: cbdv71217‐sup‐0001‐SuppMat.docx. All data generated and analyzed during this study are included in this article and Supporting Information. Further details about this related data are available upon request to the corresponding author. [file CBDV-23-e03856-s001.docx]

**Supplementary material – LC-MS/MS section**

**MSdial 4.9 Parameters**

Table S1 - Parameter settings for MS-DIAL

| **Data collection parameters** | MS1 Data type | Centroid |
| --- | --- | --- |
|  | MS2 Data type | Centroid |
|  | Ion mode | Positive |
|  | Target | Metabolomics |
|  | Retention time | 0 -18 min |
|  | Mass range | 50 -1300 |
|  | MS2 mass range | 50 -1300 |
| **Centroid parameters** | MS1 tolerance | 0.02 |
|  | MS2 tolerance | 0.06 |
| **Isotope recognition** | Maximum charged number | 2 |
| **Peak detection parameters** | Minimum peak width | 5 |
|  | Minimum peak height | 200 |
|  | Smoothing level | 3 |
|  | Mass slice width | 0.1 |
| **Deconvolution parameters** | Sigma window value | 0.5 |
|  | MS2Dec amplitude cut off | 0 |
|  | Exclude after precursor | True |
|  | Keep isotope until | 0.5 |
| **Alignment parameters** | Retention time tolerance | 0.1 |
|  | MS1 tolerance | 0.025 |
|  | Retention time factor | 0.5 |
|  | MS1 factor | 0.5 |
|  | Remove feature based on blank | True |

Table S2. Annotation of chemical components in the hydroethanolic leaves and stem bark extracts of *C. vitifolium* by UHPLC-MS/MS in positive mode.

| **Leaves** | | | | | | |
| --- | --- | --- | --- | --- | --- | --- |
| **No.** | **RT (min)** | **M+H** | **Annotated** | **Confidence level** | **Ref.** |  |
| 1 | 1.75 | 163.0602 | Glucosan | L3 | - |  |
| 2 | 1.75 | 325.1140 | GlyTouCan:G99033OS | L3 | - |  |
| 3 | 1.75 | 198.0970 | (2*R*,3*R*,4*R*,5*S*)-5-amino-6-hydroperoxyhexane-1,2,3,4-tetrol | L3 | - |  |
| 4 | 1.83 | 193.0711 | Quinic acid | L2 | ^[1]^ |  |
| 5 | 1.95 | 136.0613 | Adenin | L3 | - |  |
| 6 | 2.11 | 254.1254 | 2-*O*-*D*-glucosaminyl glycerol | L3 | - |  |
| 7 | 2.26 | 345.0831 | Theogallin | L2 | ^[2]^ |  |
| 8 | 3.20 | 166.0857 | Phenylalanine | L2 | ^[3]^ |  |
| 9 | 3.94 | 345.0830 | Theogallin | L2 | ^[2]^ |  |
| 10 | 5.75 | 497.0942 | 3,4-di-*O*-Galloylquinic acid | L2 | ^[1]^ |  |
| 11 | 6.14 | 433.1331 | Licoagroside B | L2 | ^[4]^ |  |
| 12 | 6.23 | 441.0998 | [6-(3,5-dihydroxyphenoxy)-3,4,5-trihydroxyoxan-2-yl]methyl 3,4,5-trihydroxybenzoate | L3 | - |  |
| 13 | 6.41 | 652.1155 | Corilagin | L2 | ^[5]^ |  |
| 14 | 6.54 | 595.1646 | Violantin | L2 | ^[6]^ |  |
| 15 | 6.79 | 455.1180 | [6-(2,4-dihydroxy-6-methylphenoxy)-3,4,5-trihydroxyoxan-2-yl]methyl 3,4,5-trihydroxybenzoate | L2 | ^[7]^ |  |
| 16 | 7.20 | 633.1041 | Myricetin 3-(6''-galloylgalactoside) | L2 | ^[8]^ |  |
| 17 | 7.30 | 449.1097 | Isoorientin | L2 | ^[9]^ |  |
| 18 | 7.33 | 389.2188 | Lauroside E | L2 | ^[10]^ |  |
| 19 | 7.49 | 481.1000 | Isomyricitrin | L2 | ^[11]^ |  |
| 20 | 7.86 | 617.1146 | Quercetin 3-(6''-galloylglucoside) | L2 | ^[12]^ |  |
| 21 | 7.99 | 433.1147 | Vitexin | L2 | ^[9]^ |  |
| 22 | 8.17 | 465.1031 | Hyperin | L2 | ^[9]^ |  |
| 23 | 8.60 | 601.1161 | Kaempferol 3-(6''-galloylgalactoside) | L2 | ^[1]^ |  |
| 24 | 8.60 | 435.0945 | Reinutrin | L2 | ^[13]^ |  |
| 25 | 8.70 | 769.1230 | 2-{[2-(3,4-dihydroxyphenyl)-5,7-dihydroxy-4-oxo-4*H*-chromen-3-yl]oxy}-4,5-dihydroxy-6-[(3,4,5-trihydroxybenzoyloxy)methyl]oxan-3-yl 3,4,5-trihydroxybenzoate | L3 | - |  |
| 26 | 8.75 | 585.1247 | 2-[5,7-dihydroxy-2-(4-hydroxyphenyl)-4-oxo-4*H*-chromen-8-yl]-4,5-dihydroxy-6-(hydroxymethyl)oxan-3-yl 3,4,5-trihydroxybenzoate | L3 | - |  |
| 27 | 8.85 | 449.1080 | Quercetin | L2 | ^[1]^ |  |
| 28 | 8.85 | 303.0522 | Quercetin aglycone | L2 | ^[9]^ |  |
| 29 | 8.93 | 373.2238 | Byzantionoside B | L2 | ^[14]^ |  |
| 30 | 9.12 | 419.0965 | Cyanidin 3-arabinoside | L2 | ^[15]^ |  |
| 31 | 9.29 | 525.2316 | [3,4,5-trihydroxy-6-[4-(2,6,6-trimethyl-4-oxocyclohex-2-en-1-yl)butan-2-yloxy]oxan-2-yl]methyl 3,4,5-trihydroxybenzoate | L2 | ^[16]^ |  |
| 32 | 9.59 | 287.0569 | 2'-hydroxygenistein | L2 | ^[17]^ |  |
| 33 | 9.67 | 601.1201 | 2-{[2-(3,4-dihydroxyphenyl)-5,7-dihydroxy-4-oxo-4H-chromen-3-yl]oxy}-3,5-dihydroxy-6-methyloxan-4-yl 3,4,5-trihydroxybenzoate | L2 | ^[1]^ |  |
| 34 | 9.85 | 525.2348 | [3,4,5-trihydroxy-6-[4-(2,6,6-trimethyl-4-oxocyclohex-2-en-1-yl)butan-2-yloxy]oxan-2-yl]methyl 3,4,5-trihydroxybenzoate | L2 | ^[16]^ |  |
| 35 | 9.97 | 611.1358 | Helichrysoside | L2 | ^[18]^ |  |
| 36 | 10.06 | 641.1524 | Quercetin 3-(6''-ferulylglucoside) | L2 | ^[19]^ |  |
| 37 | 10.28 | 595.1402 | (6-{[2-(3,4-dihydroxyphenyl)-5-hydroxy-4-oxo-4*H*-chromen-7-yl]oxy}-3,4,5-trihydroxyoxan-2-yl)methyl 3-(4-hydroxyphenyl)prop-2-enoate | L3 | - |  |
| 38 | 10.46 | 625.1492 | Luteolin 7-(6''-ferulylglucoside) | L2 | ^[20]^ |  |
| 39 | 10.76 | 579.1454 | Vitexin 2''-*O*-*p*-coumarate | L3 | - |  |
| 40 | 10.96 | 609.1632 | Vitexin 2''-*O*-(*E*)-ferulate | L3 | - |  |
| 41 | 12.67 | 333.0980 | 2-(3,4-dihydroxyphenyl)-5-hydroxy-3,7-dimethoxychroman-4-one | L2 | ^[9]^ |  |
| 42 | 14.28 | 289.0840 | 4*H*-naphtho(1,2-b)pyran-4-one, 6-hydroxy-2-phenyl- | L3 | - |  |
| 43 | 15.51 | 453.3384 | 3-oxooleana-1,12-diene-28-oic acid | L3 | - |  |
| 44 | 15.51 | 506.3863 | Arjunolic acid | L2 | ^[21]^ |  |
| **Stem bark** | | | | | | |
| 45 | 1.60 | 181.0710 | *D*-Glucose | L2 | ^[22]^ |  |
| 46 | 1.70 | 343.1230 | Sucrose | L2 | ^[23]^ |  |
| 47 | 1.75 | 163.0600 | 3-(2,5-dioxabicyclo[2.1.0]pentan-3-yloxy)propane-1,2-diol | L3 | - |  |
| 48 | 1.77 | 325.1140 | 4-*O*-beta-*D*-glucopyranosyl-1,2-anhydro-*D*-glucopyranose | L3 | - |  |
| 49 | 1.85 | 193.0710 | Quinic acid | L2 | ^[1]^ |  |
| 50 | 2.16 | 268.1040 | (2*R*,3*R*,4*S*,5*S*,6*R*)-6-(hydroxymethyl)-3-(3-nitropropoxy)oxane-2,4,5-triol | L3 | - |  |
| 51 | 2.70 | 171.0280 | Gallic acid | L2 | ^[1]^ |  |
| 52 | 4.80 | 199.0610 | Syringic acid | L2 | ^[24]^ |  |
| 53 | 5.73 | 467.1190 | Isoglucodistylin | L2 | ^[25]^ |  |
| 54 | 6.13 | 291.0870 | Catechin | L2 | ^[26]^ |  |
| 55 | 6.58 | 451.1220 | Sinensin | L2 | ^[27]^ |  |
| 56 | 7.04 | 467.1170 | Isoglucodistylin | L2 | ^[28]^ |  |
| 57 | 7.07 | 305.0670 | Taxifolin (dihydroquercetin) | L2 | ^[29]^ |  |
| 58 | 7.20 | 459.0880 | Epigallocatechin gallate | L2 | ^[1]^ |  |
| 59 | 7.75 | 289.0710 | Dihydrokaempferol | L2 | ^[27]^ |  |
| 60 | 8.17 | 465.1020 | Isoquercetin | L2 | ^[1]^ |  |
| 61 | 8.25 | 443.0940 | Epicatechin gallate | L2 | ^[30]^ |  |
| 62 | 8.55 | 305.0650 | Taxifolin (dihydroquercetin) | L2 | ^[29]^ |  |
| 63 | 8.88 | 435.1280 | Prunin | L2 | ^[29]^ |  |
| 64 | 9.08 | 463.0880 | 3'-mono-*O*-methylellagic acid 4-*O*-alpha-L-rhamnopyranoside | L2 | ^[31]^ |  |
| 65 | 9.40 | 587.1400 | Prunin 6''-*O*-gallate | L2 | ^[32]^ |  |
| 66 | 9.60 | 433.1100 | Cosmetin | L2 | ^[9]^ |  |
| 67 | 9.65 | 289.0710 | Dihydrokaempferol | L2 | ^[27]^ |  |
| 68 | 9.67 | 599.1140 | 8-[5,7-dihydroxy-2-(4-hydroxy-3-methoxyphenyl)-4-oxochromen-8-yl]-5,7-dihydroxy-2-(4-hydroxy-3-methoxyphenyl)chromen-4-one | L2 | ^[33]^ |  |
| 69 | 10.39 | 505.0970 | 6-((2,7-dihydroxy-8-methoxy-5,10-dioxo-5,10-dihydrochromeno[5,4,3-cde]chromen-3-yl)oxy)-4,5-dihydroxy-2-methyltetrahydro-2*H*-pyran-3-yl acetate | L3 | - |  |
| 70 | 11.88 | 273.0760 | Naringenin aglycone | L2 | ^[9]^ |  |
| 71 | 12.17 | 271.0610 | Apigenin aglycone | L2 | ^[9]^ |  |

**Leaves – Mass spectra of metabolites**

**Suppl. 1 Fig. S1**: UV, MS^1^, MS^2^ Spectra of Glucosan, peak **1**, *m/z* 163.0602 [M+H]^+^, C_6_H_10_O_5._

__

**Suppl. 1 Fig. S2**: UV, MS^1^, MS^2^ Spectra of GlyTouCan:G99033OS, peak **2**, *m/z* 325.1140 [M+H]^+^, C_12_H_20_O_10._

__

**Suppl. 1 Fig. S3**: UV, MS^1^, MS^2^ Spectra of (2*R*,3*R*,4*R*,5*S*)-5-amino-6-hydroperoxyhexane-1,2,3,4-tetrol, peak **3**, *m/z* 198.0970 [M+H]^+^, C_6_H_15_NO_6_.

**Suppl. 1 Fig. S4**: UV, MS^1^, MS^2^ Spectra of Quinic acid, peak **4,** *m/z* 193.0711[M+H]^+^, C_7_H_12_O_6._

__

**Suppl. 1 Fig. S5**: UV, MS^1^, MS^2^ Spectra of Adenin, peak **5,** *m/z* 136.0613 [M+H]^+^, C_5_H_5_N5.

**Suppl. 1 Fig. S6**: UV, MS^1^, MS^2^ Spectra of 2-O-d-glucosaminyl glycerol, peak **6,** *m/z* 254.1254 [M+H]^+^, C_9_H_19_NO_7_.

**Suppl. 1 Fig. S7**: UV, MS^1^, MS^2^ Spectra of Theogallin, peak **7,** *m/z* 345.0831 [M+H]^+^, C_14_H_16_O_10_.

**Suppl. 1 Fig. S8**: UV, MS^1^, MS^2^ Spectra of Phenylalanine, peak **8,** *m/z* 166.0857 [M+H]^+^, C_9_H_11_NO_2_.

**Suppl. 1 Fig. S9**: UV, MS^1^, MS^2^ Spectra of Theogallin, peak **9,** *m/z* 345.0830 [M+H]^+^, C_14_H_16_O_10_.

**Suppl. 1 Fig. S10**: UV, MS^1^, MS^2^ Spectra of 3,4-di-*O*-Galloylquinic Acid, peak **10,** *m/z* 497.0941 [M+H]^+^, C_21_H_20_O_14_.

**Suppl. 1 Fig. S11**: UV, MS^1^, MS^2^ Spectra of Licoagroside B, peak **11,** *m/z* 433.1331 [M+H]^+^, C_19_H_20_O_12_.

**Suppl. 1 Fig. S12**: UV, MS^1^, MS^2^ Spectra of [6-(3,5-dihydroxyphenoxy)-3,4,5-trihydroxyoxan-2-yl]methyl 3,4,5-trihydroxybenzoate, peak **12,** *m/z* 441.0998 [M+H]^+^, C_19_H_20_O_12_.

**Suppl. 1 Fig. S13**: UV, MS^1^, MS^2^ Spectra of Corilagin, peak **13,** *m/z* 652.1155 [M + H+ H2O]^+^, C_27_H_22_O_18_.

**Suppl. 1 Fig. S14**: UV, MS^1^, MS^2^ Spectra of Violantin, peak **14,** *m/z* 595.1646 [M + H+]^+^, C_27_H_30_O_15_.

**Suppl. 1 Fig. S15**: UV, MS^1^, MS^2^ Spectra of [6-(2,4-dihydroxy-6-methylphenoxy)-3,4,5-trihydroxyoxan-2-yl]methyl 3,4,5-trihydroxybenzoate, peak **15,** *m/z* 455.1180 [M + H+]^+^, C_20_H_22_O_12_.

**Suppl. 1 Fig. S16**: UV, MS^1^, MS^2^ Spectra of Myricetin 3-(6''-galloylgalactoside), peak **16,** *m/z* 633.1041 [M + H+]^+^, C_28_H_24_O_17_.

**Suppl. 1 Fig. S17**: UV, MS^1^, MS^2^ Spectra of Isoorientin, peak **17,** *m/z* 633.1041 [M + H+]^+^, C_21_H_20_O_11_.

**Suppl. 1 Fig. S18**: UV, MS^1^, MS^2^ Spectra of Lauroside E, peak **18,** *m/z* 389.2188 [M + H+]^+^, C_19_H_32_O_8_.

**Suppl. 1 Fig. S19**: UV, MS^1^, MS^2^ Spectra of Isomyricitrin, peak **19,** *m/z* 481.1000 [M + H+]^+^, C_21_H_20_O_13_.

**Suppl. 1 Fig. S20**: UV, MS^1^, MS^2^ Spectra of Quercetin 3-(6''-galloylglucoside), peak **20,** *m/z* 617.1146 [M + H+]^+^, C_28_H_24_O_16_.

**Suppl. 1 Fig. S21**: UV, MS^1^, MS^2^ Spectra of Vitexin, peak **21,** *m/z* 433.1147 [M + H+]^+^, C_21_H_20_O_10_.

**Suppl. 1 Fig. S22**: UV, MS^1^, MS^2^ Spectra of Hyperin, peak **22,** *m/z* 465.1031 [M + H+]^+^, C_21_H_20_O_12_.

**Suppl. 1 Fig. S23**: UV, MS^1^, MS^2^ Spectra of Kaempferol 3-(6''-galloylgalactoside), peak **23,** *m/z* 601.1161 [M + H+]^+^, C_28_H_24_O_15_.

**Suppl. 1 Fig. S24**: UV, MS^1^, MS^2^ Spectra of Reinutrin, peak **24,** *m/z* 435.0945 [M + H+]^+^, C_20_H_18_O_11_.

**Suppl. 1 Fig. S25**: UV, MS^1^, MS^2^ Spectra of 2-{[2-(3,4-dihydroxyphenyl)-5,7-dihydroxy-4-oxo-4*H*-chromen-3-yl]oxy}-4,5-dihydroxy-6-[(3,4,5-trihydroxybenzoyloxy)methyl]oxan-3-yl 3,4,5-trihydroxybenzoate, peak **25,** *m/z* 769.1230 [M + H+]^+^, C_35_H_28_O_20_.

**Suppl. 1 Fig. S26**: UV, MS^1^, MS^2^ Spectra of 2-[5,7-dihydroxy-2-(4-hydroxyphenyl)-4-oxo-4*H*-chromen-8-yl]-4,5-dihydroxy-6-(hydroxymethyl)oxan-3-yl 3,4,5-trihydroxybenzoate, peak **26,** *m/z* 585.1247 [M + H+]^+^, C_28_H_24_O_14_.

**Suppl. 1 Fig. S27**: UV, MS^1^, MS^2^ Spectra of Quercetin, peak **27,** *m/z* 449.1080 [M + H+]^+^, C_21_H_20_O_11_.

**Suppl. 1 Fig. S28**: UV, MS^1^, MS^2^ Spectra of Quercetin aglycone, peak **28,** *m/z* 303.0522 [M + H+]^+^, C_15_H_10_O_7_.

**Suppl. 1 Fig. S29**: UV, MS^1^, MS^2^ Spectra of Byzantionoside B, peak **29,** *m/z* 373.2238 [M + H+]^+^, C_19_H_32_O_7_.

**Suppl. 1 Fig. S30**: UV, MS^1^, MS^2^ Spectra of Cyanidin 3-arabinoside, peak **30,** *m/z* 419.0965 [M + H+]^+^, C_20_H_18_O_10_.

**Suppl. 1 Fig. S31**: UV, MS^1^, MS^2^ Spectra of [3,4,5-trihydroxy-6-[4-(2,6,6-trimethyl-4-oxocyclohex-2-en-1-yl)butan-2-yloxy]oxan-2-yl]methyl 3,4,5-trihydroxybenzoate, peak **31,** *m/z* 525.2316 [M + H+]^+^, C_26_H_36_O_11_.

**Suppl. 1 Fig. S32**: UV, MS^1^, MS^2^ Spectra of 2'-hydroxygenistein, peak **32,** *m/z* 287.0569 [M + H+]^+^, C_15_H_10_O_6_.

**Suppl. 1 Fig. S33**: UV, MS^1^, MS^2^ Spectra of 2-{[2-(3,4-dihydroxyphenyl)-5,7-dihydroxy-4-oxo-4H-chromen-3-yl]oxy}-3,5-dihydroxy-6-methyloxan-4-yl 3,4,5-trihydroxybenzoate, peak **33,** *m/z* 601.1201 [M + H+]^+^, C_28_H_24_O_15_.

**Suppl. 1 Fig. S34**: UV, MS^1^, MS^2^ Spectra of [3,4,5-trihydroxy-6-[4-(2,6,6-trimethyl-4-oxocyclohex-2-en-1-yl)butan-2-yloxy]oxan-2-yl]methyl 3,4,5-trihydroxybenzoate, peak **34,** *m/z* 525.2348 [M + H+]^+^, C_26_H_36_O_11_.

**Suppl. 1 Fig. S35**: UV, MS^1^, MS^2^ Spectra of Helichrysoside, peak **35,** *m/z* 611.1358 [M + H+]^+^, C_30_H_26_O_14_.

**Suppl. 1 Fig. S36**: UV, MS^1^, MS^2^ Spectra of Quercetin 3-(6''-ferulylglucoside), peak **36,** *m/z* 641.1524 [M + H+]^+^, C_31_H_28_O_15_.

**Suppl. 1 Fig. S37**: UV, MS^1^, MS^2^ Spectra of (6-{[2-(3,4-dihydroxyphenyl)-5-hydroxy-4-oxo-4H-chromen-7-yl]oxy}-3,4,5-trihydroxyoxan-2-yl)methyl 3-(4-hydroxyphenyl)prop-2-enoate, peak **37,** *m/z* 595.1402 [M + H+]^+^, C_30_H_26_O_13_.

**Suppl. 1 Fig. S38**: UV, MS^1^, MS^2^ Spectra of Luteolin 7-(6''-ferulylglucoside), peak **38,** *m/z* 625.1492 [M + H+]^+^, C_31_H_28_O_14_.

**Suppl. 1 Fig. S39**: UV, MS^1^, MS^2^ Spectra of Vitexin 2''-*O*-*p*-coumarate, peak **39,** *m/z* 579.1454 [M + H+]^+^, C_30_H_26_O_12_.

**Suppl. 1 Fig. S40**: UV, MS^1^, MS^2^ Spectra of Vitexin 2''-*O*-(*E*)-Ferulate, peak **40,** *m/z* 609.1632 [M + H+]^+^, C_31_H_28_O_13_.

**Suppl. 1 Fig. S41**: UV, MS^1^, MS^2^ Spectra of 2-(3,4-dihydroxyphenyl)-5-hydroxy-3,7-dimethoxy-2,3-dihydrochromen-4-one, peak **41,** *m/z* 333.0980 [M + H+]^+^, C_17_H_16_O_7_.

**Suppl. 1 Fig. S42**: UV, MS^1^, MS^2^ Spectra 4*H*-Naphtho(1,2-b)pyran-4-one, 6-hydroxy-2-phenyl-, peak **42,** *m/z* 289.0840 [M + H+]^+^, C_19_H_12_O_3_.

**Suppl. 1 Fig. S43**: UV, MS^1^, MS^2^ Spectra 3-Oxooleana-1,12-diene-28-oic acid, peak **43,** *m/z* 453.3384 [M + H+]^+^, C_30_H_44_O_3_.

**Suppl. 1 Fig. S44**: UV, MS^1^, MS^2^ Spectra Arjunolic Acid, peak **44,** *m/z* 506.3863 [M + H+ H_2_O]^+^, C_30_H_48_O_5_.

**Stem bark -** **Mass spectra of metabolites**

**Suppl. 1 Fig. S45**: UV, MS^1^, MS^2^ Spectra *D*-Glucose, peak **45,** *m/z* 181.0710 [M + H+]^+^, C_6_H_12_O_6_.

**Suppl. 1 Fig. S46**: UV, MS^1^, MS^2^ Spectra Sucrose, peak **46,** *m/z* 343.1230 [M + H]^+^, C_12_H_22_O_11_.

**Suppl. 1 Fig. S47**: UV, MS^1^, MS^2^ Spectra 3-(2,5-Dioxabicyclo[2.1.0]pentan-3-yloxy)propane-1,2-diol, peak **47,** *m/z* 163.0600 [M + H+]^+^, C_6_H_10_O_5_.

**Suppl. 1 Fig. S48**: UV, MS^1^, MS^2^ Spectra 4-*O*-beta-*D*-Glucopyranosyl-1,2-anhydro-*D*-glucopyranose, peak **48,** *m/z* 325.1140 [M + H+]^+^, C_12_H_20_O_10_.

**Suppl. 1 Fig. S49**: UV, MS^1^, MS^2^ Spectra Quinic Acid, peak **49,** *m/z* 193.0710 [M + H+]^+^, C_7_H_12_O_6_.

__

**Suppl. 1 Fig. S50**: UV, MS^1^, MS^2^ Spectra (2*R*,3*R*,4*S*,5*S*,6*R*)-6-(hydroxymethyl)-3-(3-nitropropoxy)oxane-2,4,5-triol, peak **50,** *m/z* 268.1040 [M + H+]^+^, C_9_H_17_NO_8_.

**Suppl. 1 Fig. S51**: UV, MS^1^, MS^2^ Spectra Gallic Acid, peak **51,** *m/z* 171.0280 [M + H+]^+^, C_7_H_6_O_5_.

**Suppl. 1 Fig. S52**: UV, MS^1^, MS^2^ Spectra Syringic acid, peak **52,** *m/z* 199.0610 [M + H+]^+^, C_9_H_10_O_5_.

**Suppl. 1 Fig. S53**: UV, MS^1^, MS^2^ Spectra Isoglucodistylin, peak **53,** *m/z* 467.1190 [M + H+]^+^, C_21_H_22_O_12_.

**Suppl. 1 Fig. S54**: UV, MS^1^, MS^2^ Spectra Catechin, peak **54,** *m/z* 291.0870 [M + H+]^+^, C_15_H_14_O_6_.

**Suppl. 1 Fig. S55**: UV, MS^1^, MS^2^ Spectra Sinensin, peak **55,** *m/z* 451.1220 [M + H+]^+^, C_21_H_22_O_11_.

**Suppl. 1 Fig. S56**: UV, MS^1^, MS^2^ Spectra Isoglucodistylin, peak **56,** *m/z* 467.1170 [M + H+]^+^, C_21_H_22_O_12_.

**Suppl. 1 Fig. S57**: UV, MS^1^, MS^2^ Spectra Taxifolin (dihydroquercetin), peak **57,** *m/z* 305.0670 [M + H+]^+^, C_15_H_12_O_7_.

**Suppl. 1 Fig. S58**: UV, MS^1^, MS^2^ Spectra Epigallocatechin gallate, peak **58,** *m/z* 459.0880 [M + H+]^+^, C_22_H_18_O_11_.

**Suppl. 1 Fig. S59**: UV, MS^1^, MS^2^ Spectra Dihydrokaempferol, peak **59,** *m/z* 289.0710 [M + H+]^+^, C_15_H_12_O_6_.

**Suppl. 1 Fig. S60**: UV, MS^1^, MS^2^ Spectra Isoquercetin, peak **60,** *m/z* 465.1020 [M + H+]^+^, C_21_H_20_O_12_.

**Suppl. 1 Fig. S61**: UV, MS^1^, MS^2^ Spectra Epicatechin gallate, peak **61,** *m/z* 443.0940 [M + H+]^+^, C_22_H_18_O_10_.

**Suppl. 1 Fig. S62**: UV, MS^1^, MS^2^ Spectra Taxifolin (dihydroquercetin), peak **62,** *m/z* 305.0650 [M + H+]^+^, C_15_H_12_O_7_.

**Suppl. 1 Fig. S63**: UV, MS^1^, MS^2^ Spectra Prunin, peak **63,** *m/z* 435.1280 [M + H+]^+^, C_21_H_22_O_10_.

**Suppl. 1 Fig. S64**: UV, MS^1^, MS^2^ Spectra 3'-Mono-*O*-Methylellagic Acid 4-*O*-alpha-*L*-Rhamnopyranoside, peak **64,** *m/z* 463.0880 [M + H+]^+^, C_21_H_18_O_12_.

**Suppl. 1 Fig. S65**: UV, MS^1^, MS^2^ Spectra Prunin 6''-*O*-Gallate, peak **65,** *m/z* 587.1400 [M + H+]^+^, C_28_H_26_O_14_.

**Suppl. 1 Fig. S66**: UV, MS^1^, MS^2^ Spectra Cosmetin, peak **66,** *m/z* 433.1100 [M + H+]^+^, C_21_H_20_O_10_.

**Suppl. 1 Fig. S67**: UV, MS^1^, MS^2^ Spectra Dihydrokaempferol, peak **67,** *m/z* 289.0710 [M + H+]^+^, C_15_H_12_O_6_.

**Suppl. 1 Fig. S68**: UV, MS^1^, MS^2^ Spectra 8-[5,7-dihydroxy-2-(4-hydroxy-3-methoxyphenyl)-4-oxochromen-8-yl]-5,7-dihydroxy-2-(4-hydroxy-3-methoxyphenyl)chromen-4-one, peak **68,** *m/z* 599.1140 [M + H+]^+^, C_32_H_22_O_12_.

**Suppl. 1 Fig. S69**: UV, MS^1^, MS^2^ Spectra 6-((2,7-dihydroxy-8-methoxy-5,10-dioxo-5,10-dihydrochromeno[5,4,3-cde]chromen-3-yl)oxy)-4,5-dihydroxy-2-methyltetrahydro-2*H*-pyran-3-yl acetate, peak **69,** *m/z* 505.0970 [M + H+]^+^, C_23_H_20_O_13_.

**Suppl. 1 Fig. S70**: UV, MS^1^, MS^2^ Spectra Naringenin aglycone, peak **70,** *m/z* 273.0760 [M + H+]^+^, C_15_H_12_O_5_.

**Suppl. 1 Fig. S71**: UV, MS^1^, MS^2^ Spectra Apigenin aglycone, peak **71,** *m/z* 271.0601 [M + H+]^+^, C_15_H_10_O_5_.

References

[1] F. Galvão, E. dos Santos, F. Gomes da Silva Dantas, J. Irlan da Silva Santos, T. da Paz Costa Sauda, A. Carvalho dos Santos, R. I. Carvalho Souza, L. da Silva Pinto, C. A. Ferreira Moraes, A. Sangalli, C. A. Leite Kassuya, C. R. Nogueira, K. M. Pires de Oliveira, *Journal of Ethnopharmacology* **2023**, *302*.

[2] D. Qi, J. Li, X. Qiao, M. Lu, W. Chen, A. Miao, W. Guo, C. Ma, *Journal of Agricultural and Food Chemistry* **2019**, *67*, 6672–6682.

[3] M. V. V. Lyrio, D. G. Debona, A. E. Feu, N. A. dos Santos, A. d. S. Gonçalves, R. M. Kuster, E. V. R. de Castro, W. Romão, *Journal of the American Society for Mass Spectrometry* **2025**, *36*, 1213–1226.

[4] R. Gupta, C. W. Min, K. Kramer, G. K. Agrawal, R. Rakwal, K. H. Park, Y. Wang, I. Finkemeier, S. T. Kim, *Proteomics* **2018**, *18*.

[5] B. Fogliani, P. Raharivelomanana, J.-P. Bianchini, S. Bouraı¨ma-Madjèbi, E. Hnawia, *Phytochemistry* **2005**, *66*, 241–247.

[6] L. M. Casanova, W. Gu, S. S. Costa, P. B. Jeppesen, *Journal of Natural Products* **2017**, *80*, 3267–3275.

[7] Y. P. Zou, C. H. Tan, B. D. Wang, D. Y. Zhu, S. K. Kim, *Helvetica Chimica Acta* **2008**, *91*, 2168–2173.

[8] L.-C. Lin, C.-J. Chou, *Planta Medica* **2000**, *66*, 382–383.

[9] N. Samba, A. M. Barrios, E. G. De León, C. Raposo, R. A. Lahlou, J. Curto, J. M. Rodilla, A. M. Roncero, D. Diez, L. Silva, *Molecules* **2025**, *30*.

[10] K. Matsunami, I. Takamori, T. Shinzato, M. Aramoto, K. Kondo, H. Otsuka, Y. Takeda, *Chemical and Pharmaceutical Bulletin* **2006**, *54*, 1403–1407.

[11] L. Pawłowska, *Acta Societatis Botanicorum Poloniae* **2014**, *51*, 413–421.

[12] J. S. Yu, M. Park, C. Pang, L. Rashan, W. H. Jung, K. H. Kim, *Journal of Natural Products* **2020**, *83*, 2261–2268.

[13] D. M. Amaya-Cruz, I. F. Pérez-Ramírez, J. Delgado-García, C. Mondragón-Jacobo, A. Dector-Espinoza, R. Reynoso-Camacho, *Food Chemistry* **2019**, *278*, 568–578.

[14] F. M. Harraz, H. M. Hammoda, A. El-Hawiet, M. M. Radwan, A. S. Wanas, A. M. Eid, M. A. ElSohly, *Natural Product Research* **2018**, *34*, 816–822.

[15] N. P. Seeram, L. S. Adams, M. L. Hardy, D. Heber, *Journal of Agricultural and Food Chemistry* **2004**, *52*, 2512–2517.

[16] T. K. Q. Ha, T. P. Doan, H. T. T. Pham, N. H. Nguyen, T. T. Nguyen, T. B. H. Bui, *Chemical Papers* **2021**, *75*, 5323–5337.

[17] Y. H. Seo, J.-H. Jeon, M. Jeong, S. M. Ryu, W. K. Jeon, D. S. Jang, S. H. Shim, D. Lee, J.-H. Choi, J. Lee, *Journal of Natural Products* **2018**, *81*, 1598–1603.

[18] R. Hendra, P. A. Keller, *Journal of Natural Products* **2017**, *80*, 2141–2145.

[19] G. J. Niemann, *Phytochemistry* **1975**, *14*, 1437–1438.

[20] Y. Tian, L.-M. Sun, X.-Q. Liu, B. Li, Q. Wang, J.-X. Dong, *Fitoterapia* **2010**, *81*, 799–802.

[21] B. Diallo, M. Vanhaelen, R. Vanhaelen-Fastré, T. Konoshima, M. Kozuka, H. Tokuda, *Journal of Natural Products* **2004**, *52*, 879–881.

[22] M. Witting, J. Hastings, N. Rodriguez, C. J. Joshi, J. P. N. Hattwell, P. R. Ebert, M. van Weeghel, A. W. Gao, M. J. O. Wakelam, R. H. Houtkooper, A. Mains, N. Le Novère, S. Sadykoff, F. Schroeder, N. E. Lewis, H.-J. Schirra, C. Kaleta, O. Casanueva, *Frontiers in Molecular Biosciences* **2018**, *5*.

[23] E. Hernández-García, A. García, E. Garza-González, F. G. Avalos-Alanís, V. M. Rivas-Galindo, J. Rodríguez-Rodríguez, V. M. Alcantar-Rosales, C. Delgadillo-Puga, M. del Rayo Camacho-Corona, *Journal of Ethnopharmacology* **2019**, *230*, 74–80.

[24] J. K. Reinhardt, A. M. Zimmermann-Klemd, O. Danton, M. Smieško, C. Gründemann, M. Hamburger, *Journal of Natural Products* **2020**, *83*, 3012–3020.

[25] L.-M. Yang Kuo, L.-J. Zhang, H.-T. Huang, Z.-H. Lin, C.-C. Liaw, H.-L. Cheng, K.-H. Lee, S. L. Morris-Natschke, Y.-H. Kuo, H.-O. Ho, *Journal of Natural Products* **2013**, *76*, 580–587.

[26] S. González-Manzano, A. González-Paramás, C. Santos-Buelga, M. Dueñas, *Journal of Agricultural and Food Chemistry* **2009**, *57*, 1231–1238.

[27] S. Solon, C. A. Carollo, L. F. G. Brandão, C. d. S. d. Macedo, A. Klein, C. A. Dias-Junior, J. M. d. Siqueira, *Química Nova* **2012**, *35*, 1169–1172.

[28] N. Ishikura, *Agricultural and Biological Chemistry* **1982**, *46*, 1705–1706.

[29] A. B. Aguilar-Guadarrama, M. Y. Rios, *Molecules* **2018**, *23*.

[30] C. A. Schmidt, R. Murillo, T. Bruhn, G. Bringmann, M. Goettert, B. Heinzmann, V. Brecht, S. A. Laufer, I. Merfort, *Journal of Natural Products* **2010**, *73*, 2035–2041.

[31] P. Tuchinda, J. Kornsakulkarn, M. Pohmakotr, P. Kongsaeree, S. Prabpai, C. Yoosook, J. Kasisit, C. Napaswad, S. Sophasan, V. Reutrakul, *Journal of Natural Products* **2008**, *71*, 655–663.

[32] Y.-J. Zhang, T. Abe, T. Tanaka, C.-R. Yang, I. Kouno, *Chemical and Pharmaceutical Bulletin* **2002**, *50*, 841–843.

[33] T. M. Kalenga, M. M. Ndoile, Y. Atilaw, P. J. Gilissen, J. J. E. Munissi, A. Rudenko, C. Bourgard, P. Sunnerhagen, S. S. Nyandoro, M. Erdelyi, *Journal of Natural Products* **2021**, *84*, 364–372.

**Supplementary material – GC-MS section**

**LEAVES**

Table S3 - Compounds identified in the hydroethanolic leaves extract of *C. vitifolium* by GC-MS analysis.

| Peak | RT | Compounds | % Area | KI_e_ | KI_n_ | KI_l_ | Ref. |
| --- | --- | --- | --- | --- | --- | --- | --- |
| 1 | 12.038 | Dihydroxyacetone, 2TMS | 1.15 | 1216 | NF | NF |  |
| 2 | 12.503 | Benzoic acid, TMS | 40.45 | 1235 | 1242 | NF |  |
| 3 | 13.469 | Glycerol, 3TMS | 0.72 | 1273 | 1265 | 1279 | ^1^ |
| 4 | 14.798 | Glyceric acid, 3TMS | 0.33 | 1326 | 1318 | 1334 | ^2^ |
| 5 | 19.207 | Pyrogallol, 3TMS | 0.18 | 1527 | 1537 | 1535 | ^3^ |
| 6 | 19.896 | 2,3,4-Trihydroxybutyric acid tetrakis(trimethylsilyl) | 0.73 | 1562 | NF | NF |  |
| 7 | 20.893 | *D*-(-)-Ribofuranose, tetrakis (trimethylsilyl) ether | 0.08 | 1611 | NF | 1614 | ^3^ |
| 8 | 21.441 | *D*-(+)-Ribono-1,4-lactone (*R*,*S*,*R*)-, 3TMS | 0.13 | 1641 | NF | 1639 | ^1^ |
| 9 | 24.005 | *D*-Allofuranose, pentakis (trimethylsilyl) ether | 7.91 | 1781 | NF | NF |  |
| 10 | 24.29 | Methyl α-*D*-glucofuranoside, 4TMS | 2.97 | 1796 | NF | 1804 | ^2^ |
| 11 | 24.633 | *D*-(-)-Fructofuranose, pentakis (trimethylsilyl) ether | 3.87 | 1816 | NF | 1816 | ^4^ |
| 12 | 24.887 | *D*-(-)-Tagatofuranose, pentakis (trimethylsilyl) ether | 0.38 | 1832 | NF | NF |  |
| 13 | 25.026 | *β*-*D*-(+)-Talopyranose, 5TMS | 3.22 | 1840 | NF | NF |  |
| 14 | 25.105 | Methyl *α*-*D*-glucofuranoside, 4TMS | 7.13 | 1845 | NF | NF |  |
| 15 | 25.19 | *D*-(+)-Talofuranose, pentakis (trimethylsilyl) ether | 3.32 | 1850 | NF | 1842 | ^4^ |
| 16 | 25.441 | Quininic acid (5TMS) | 10.36 | 1864 | 1863 | 1875 | ^2^ |
| 17 | 25.907 | *D*-Fructose, 5TMS | 1.49 | 1891 | NF | 1881 | ^5^ |
| 18 | 26.09 | *α*-*D*-(+)-Talopyranose, 5TMS | 4.20 | 1902 | NF | NF |  |
| 19 | 26.816 | Gallic acid, 4TMS | 9.80 | 1947 | 1962 | 1954 | ^3^ |
| 20 | 27.431 | *β*-*D*-Allopyranose, 5TMS | 0.78 | 1985 | NF | NF |  |
| 21 | 27.822 | Palmitic acid, TMS | 0.36 | 2009 | 2015 | NF | ^6^ |
| 22 | 30.742 | Stearic acid, TMS | 0.08 | 2202 | 2207 | NF | ^6^ |
| 23 | 44.548 | Stigmast-5-ene, 3*β*- (trimethylsiloxy)-, (24*S*)- | 0.36 | 3288 | NF | NF | ^7^ |

**Leaves – Mass spectra of metabolites**

**Suppl 1. Fig. S72:** Mass spectrum of Dihydroxyacetone, 2TMS, peak 1.


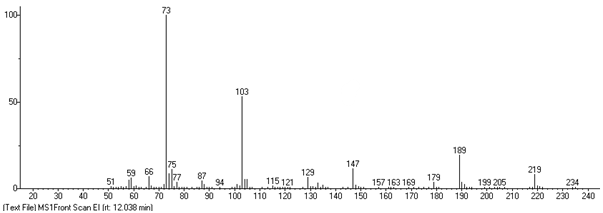


**Suppl 1. Fig. S73:** Mass spectrum of Benzoic Acid, TMS, peak 2.


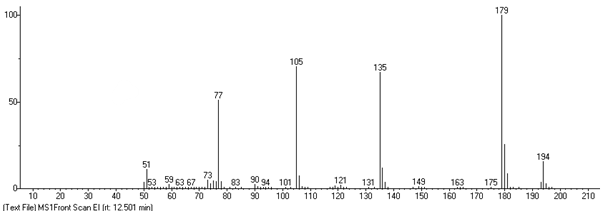


**Suppl 1. Fig. S74:** Mass spectrum of Glycerol, 3TMS, peak 3.


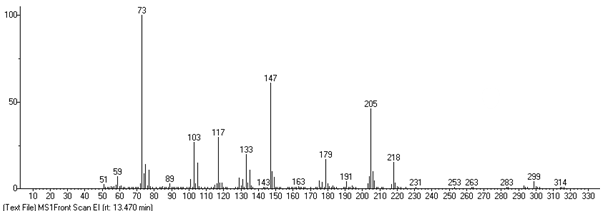


**Suppl 1. Fig. S75:** Mass spectrum of Glyceric acid, 3TMS, peak 4.


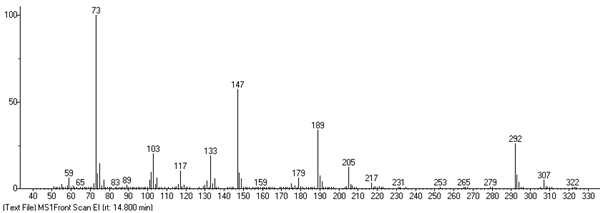


**Suppl 1. Fig. S76:** Mass spectrum of Pyrogallol, 3TMS, peak 5.


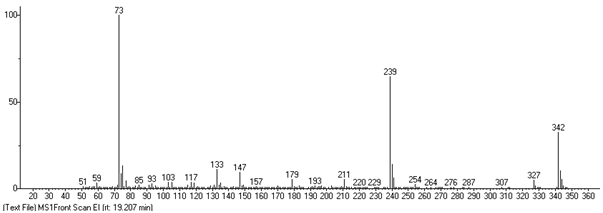


**Suppl 1. Fig. S77:** Mass specturm of 2,3,4-trihydroxybutyric acid tetrakis(trimethylsilyl), peak 6.


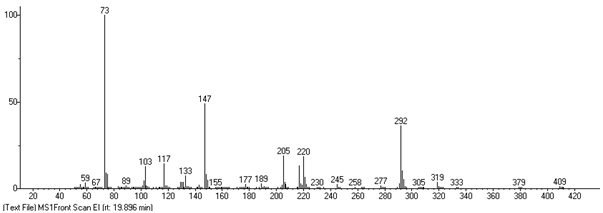


**Suppl 1. Fig. S78:** Mass spectrum of *D*-(-)-Ribofuranose, tetrakis (trimethylsilyl) ether, peak 7.


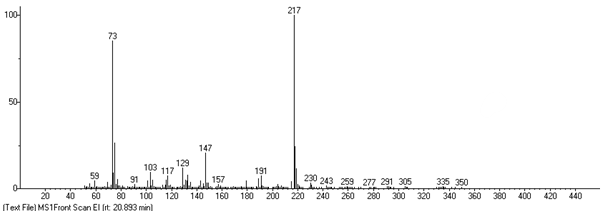


**Suppl 1. Fig. S79:** Mass spectrum of *D*-(+)-Ribono-1,4-lactone (*R*,*S*,*R*)-, 3TMS, peak 8.


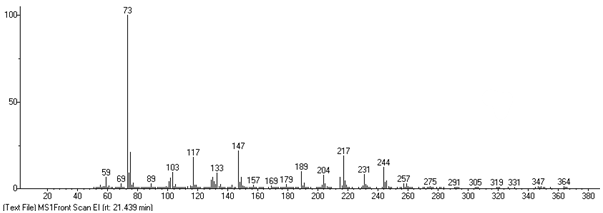


**Suppl 1. Fig. S80:** Mass spectrum of *D*-Allofuranose, pentakis (trimethylsilyl) ether, peak 9.


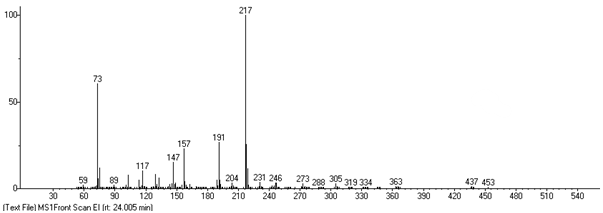


**Suppl 1. Fig. S81:** Mass spectrum of Methyl *α*-*D*-glucofuranoside, 4TMS, peak 10.


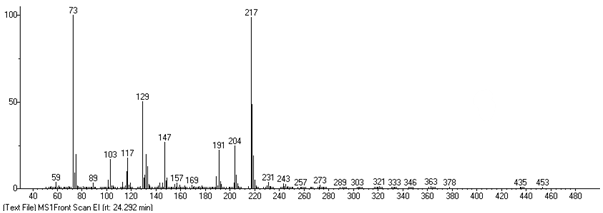


**Suppl 1. Fig. S82:** Mass spectrum of *D*-(-)-Fructofuranose, pentakis (trimethylsilyl) ether, peak 11.


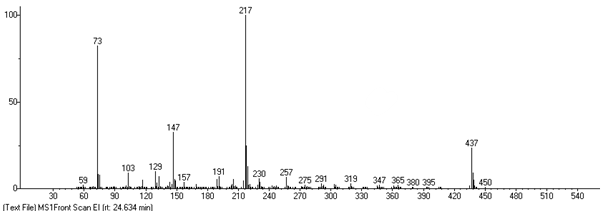


**Suppl 1. Fig. S83:** Mass spectrum of *D*-(-)-Tagatofuranose, pentakis (trimethylsilyl) ether, peak 12.


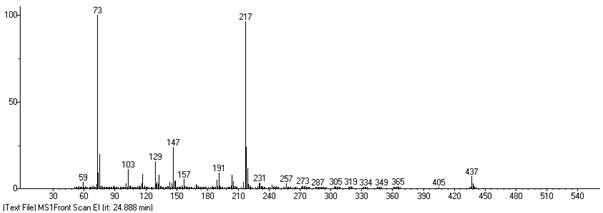


**Suppl 1. Fig. S84:** Mass spectrum of *β*-*D*-(+)-Talopyranose, 5TMS, peak 13.


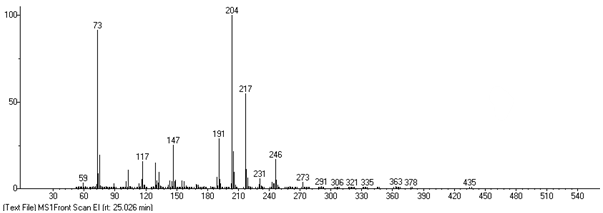


**Suppl 1. Fig. S85:** Mass spectrum of Methyl *α*-*D*-glucofuranoside, 4TMS, peak 14.


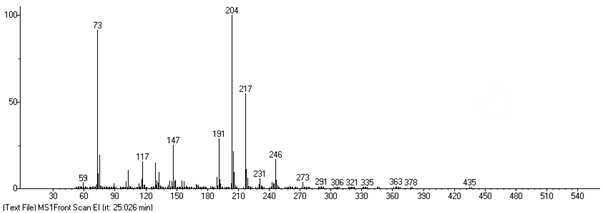


**Suppl 1. Fig. S86:** Mass spectrum of *D*-(+)-Talofuranose, pentakis (trimethylsilyl) ether, peak 15.


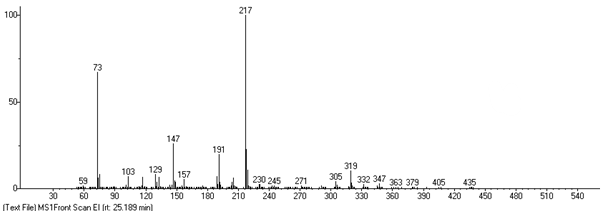


**Suppl 1. Fig. S87:** Mass spectrum of Quininic acid (5TMS), peak 16.


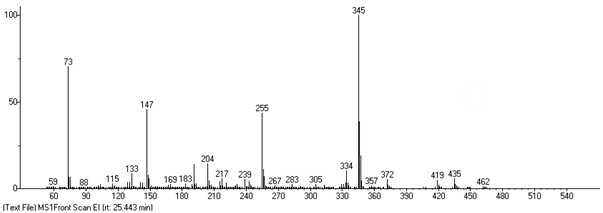


**Suppl 1. Fig. S88:** Mass spectrum of *D*-Fructose, 5TMS, peak 17.


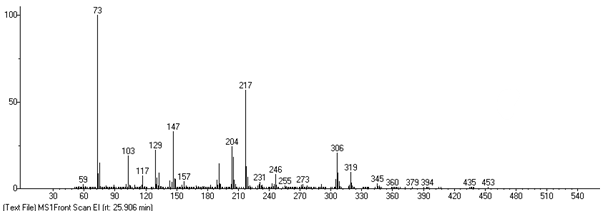


**Suppl 1. Fig. S89:** Mass spectrum of *α*-*D*-(+)-Talopyranose, 5TMS, peak 18.


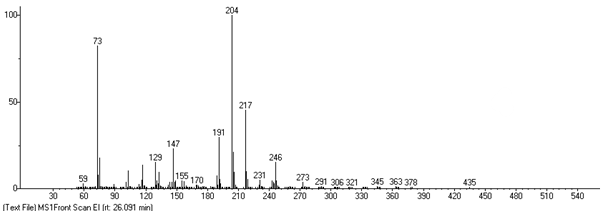


**Suppl 1. Fig. S90:** Mass spectrum of Gallic acid, 4TMS, peak 19.


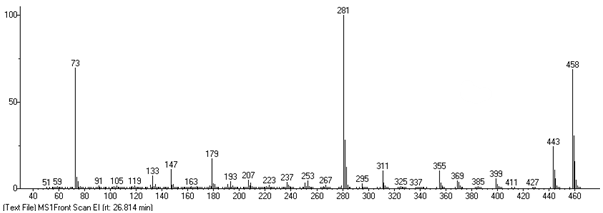


**Suppl 1. Fig. S91:** Mass spectrum of *β*-*D*-Allopyranose, 5TMS, peak 20.


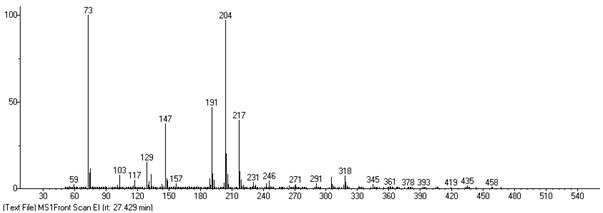


**Suppl 1. Fig. S92:** Mass spectrum of Palmitic Acid, TMS, peak 21.


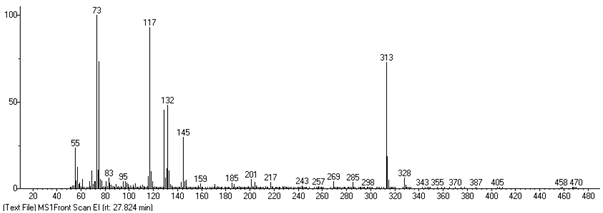


**Suppl 1. Fig. S93:** Mass spectrum of Stearic acid, TMS, peak 22.


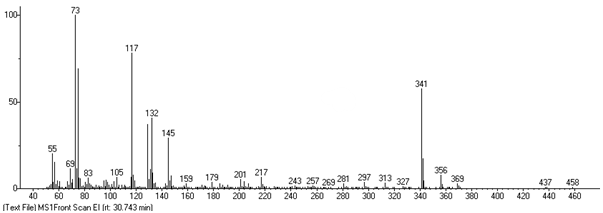


**Suppl 1. Fig. S94:** Mass spectrum of Stigmast-5-ene, 3*β*-(trimethylsiloxy)-, (24*S*)-, peak 23.


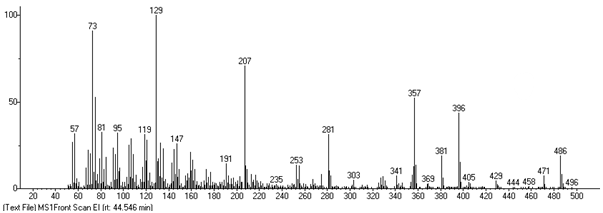


**STEM BARK**

Table S4 - Compounds identified in the hydroethanolic stem bark extract of C. vitifolium by GC-MS analysis.

| Peak | RT | Compounds | Mass | % Area | KIe | KIn | KIl | Ref. |
| --- | --- | --- | --- | --- | --- | --- | --- | --- |
| 1 | 12.496 | Benzoic acid, TMS | 194.07 | 32.61 | 1235 | 1242 | NF |  |
| 2 | 13.472 | Glycerol, 3TMS | 308.16 | 3.37 | 1273 | 1265 | 1279 | ^1^ |
| 3 | 14.796 | Glyceric acid, 3TMS | 322.14 | 0.20 | 1326 | 1318 | 1334 | ^2^ |
| 4 | 15.608 | Erythrono-1,4-lactone, (*E*)-, 2TMS | 262.10 | 0.11 | 1362 | NF | NF |  |
| 5 | 18.251 | Malic acid, 3TMS | 350.14 | 0.18 | 1481 | 1479 | 1481 | ^4^ |
| 6 | 18.823 | *L*-Threitol 4TMS | 410.21 | 0.11 | 1507 | 1491 | 1509 | ^4^ |
| 7 | 19.897 | 2,3,4-Trihydroxybutyric acid tetrakis(trimethylsilyl) | 424.19 | 0.93 | 1562 | NF | NF |  |
| 8 | 21.441 | *D*-(+)-Ribono-1,4-lactone (*R*,*S*,*R*)-, 3TMS | 364.15 | 0.11 | 1641 | NF | 1639 | ^4^ |
| 9 | 22.044 | Arabinofuranose, 1,2,3,5-tetrakis-*O*-(trimethylsilyl) | 438.21 | 0.11 | 1673 | NF | NF |  |
| 10 | 23.999 | *D*-Allofuranose, pentakis (trimethylsilyl) ether | 540.26 | 4.45 | 1780 | NF | NF |  |
| 11 | 24.284 | Methyl *α*-*D*-glucofuranoside, 4TMS | 482.23 | 2.88 | 1796 | NF | 1804 | ^2^ |
| 12 | 24.638 | *D*-(-)-Fructofuranose, pentakis (trimethylsilyl) ether | 540.26 | 5.97 | 1817 | NF | 1816 | ^4^ |
| 13 | 24.801 | *D*-(+)-Talofuranose, pentakis (trimethylsilyl) ether | 540.26 | 0.36 | 1826 | 1822 | 1828 | ^1^ |
| 14 | 24.881 | *D*-(-)-Ribofuranose, tetrakis (trimethylsilyl) ether | 438.21 | 0.39 | 1831 | NF | 1826 | ^1^ |
| 15 | 25.03 | *β*-*D*-(+)-Talopyranose, 5TMS | 540.26 | 3.04 | 1840 | NF | 1852 | ^1^ |
| 16 | 25.465 | Quininic acid (5TMS) | 552.26 | 14.61 | 1866 | 1863 | 1875 | ^1^ |
| 17 | 25.754 | *L*-Glucono-1,4 lactone (2*S*,3*R*,4*S*,5*S*)-,4TMS | 466.20 | 0.98 | 1882 | NF | NF |  |
| 18 | 25.947 | *β*-*D*-(+)-Mannopyranose, 5TMS | 540.26 | 2.17 | 1893 | NF | 1888 | ^3^ |
| 19 | 26.096 | *α*-*D*-Allopyranose, 5TMS | 540.26 | 4.33 | 1902 | NF | NF |  |
| 20 | 26.145 | *D*-Altrose, 5TMS | 540.26 | 1.43 | 1905 | 1911 | NF |  |
| 21 | 26.779 | Gallic acid, 4TMS | 458.17 | 1.51 | 1945 | NF | 1954 | ^3^ |
| 22 | 27.432 | *β*-*D*-Allopyranose, 5TMS | 540.26 | 1.14 | 1985 | NF | NF |  |
| 23 | 27.752 | *D*-Gluconic acid, 6TMS | 628.29 | 0.39 | 2005 | 1997 | NF |  |
| 24 | 27.823 | Palmitic acid, TMS | 328.27 | 0.21 | 2009 | 2015 | NF | ^6^ |
| 25 | 35.346 | Sucrose, 8TMS | 918.43 | 11.94 | 2543 | NF | 2543 | ^8^ |
| 26 | 36.388 | *D*-(+)-Turanose, octakis (trimethylsilyl) ether | 918.43 | 0.41 | 2626 | NF | NF |  |
| 27 | 36.723 | Lactose, 8TMS | 918.43 | 2.89 | 2653 | NF | NF |  |
| 28 | 38.379 | 3-*α*-Mannobiose, octakis (trimethylsilyl) ether | 918.43 | 0.92 | 2792 | NF | NF |  |
| 29 | 38.798 | *D*-Lactose, 8TMS | 918.4 | 0.51 | 2824 | NF | NF |  |
| 30 | 40.81 | Galactinol, nonakis (trimethylsilyl) ether | 194.07 | 0.46 | 3008 | 2946 | NF |  |

**Stem bark – Mass spectra of metabolites**

**Suppl 1. Fig. S95:** Mass spectrum of Benzoic acid, TMS, peak 1.


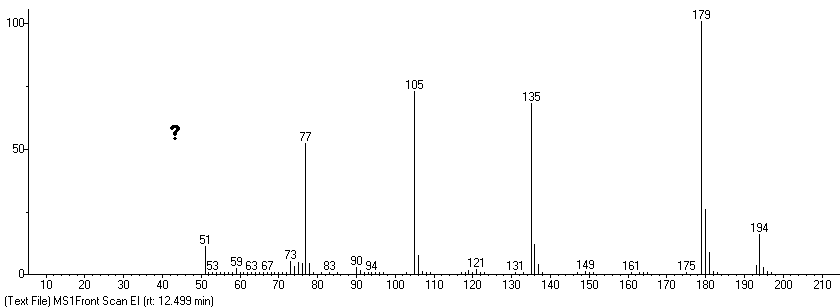

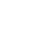


**Suppl 1. Fig. S96**: Mass spectrum of Glycerol, 3TMS, peak 2.


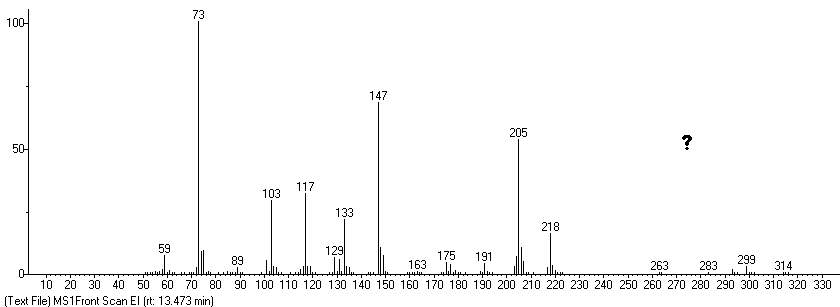

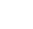


**Suppl 1. Fig. S97:** Mass spectrum of Glyceric acid, 3TMS, peak 3.


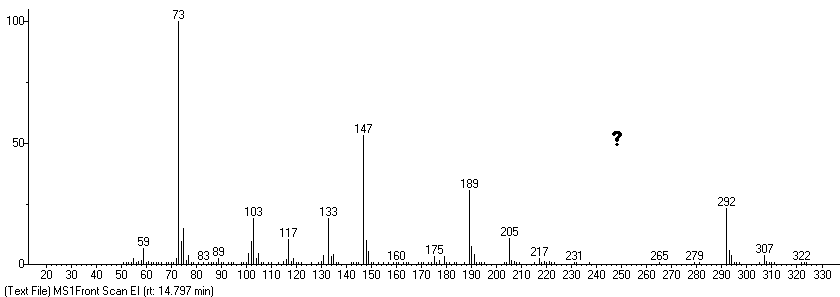

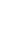


**Suppl 1. Fig. S98:** Mass spectrum of Erythrono-1,4-lactone, (*E*)-, 2TMS, peak 4.


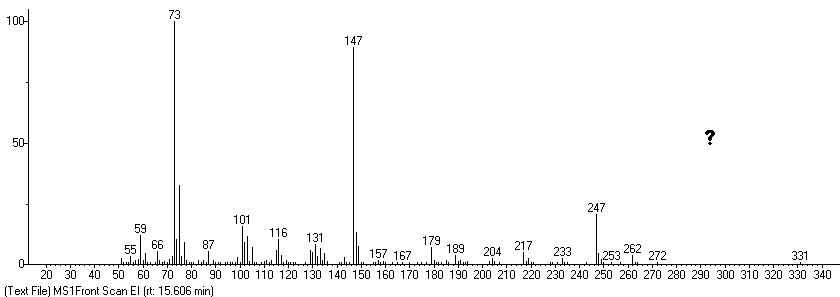

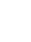


**Suppl 1. Fig. S99:** Mass spectrum of Malic acid, 3TMS, peak 5.


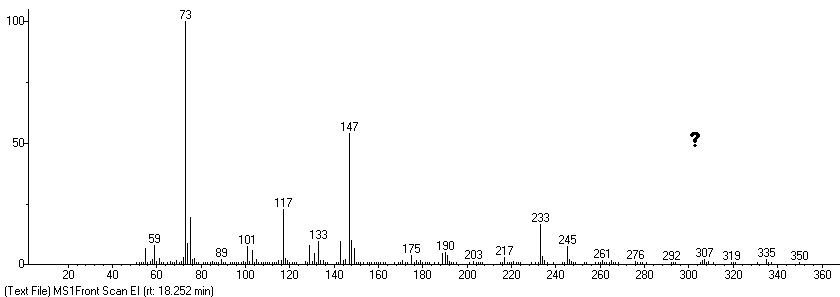

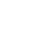


**Suppl 1. Fig. S100:** Mass spectrum of *L*-Threitol, 4TMS, peak 6.


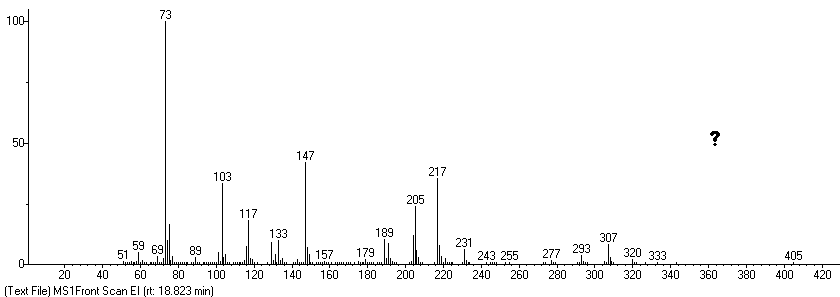

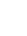


**Suppl 1. Fig. S101:** Mass spectrum of 2,3,4-trihydroxybutyric acid tetrakis(trimethylsilyl), peak 7.


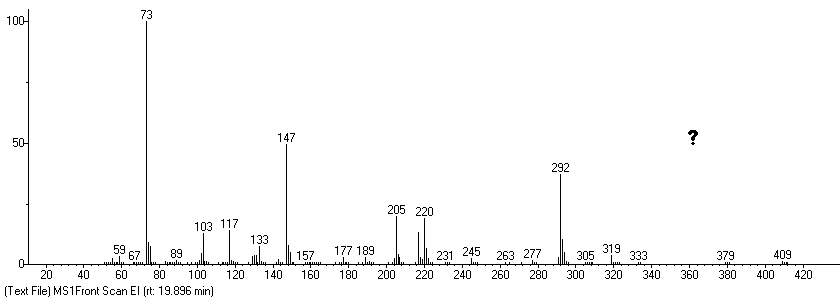

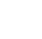


**Suppl 1. Fig. S102:** Mass spectrum of *D*-(+)-Ribono-1,4-lactone (*R*,*S*,*R*)-, 3TMS, peak 8.


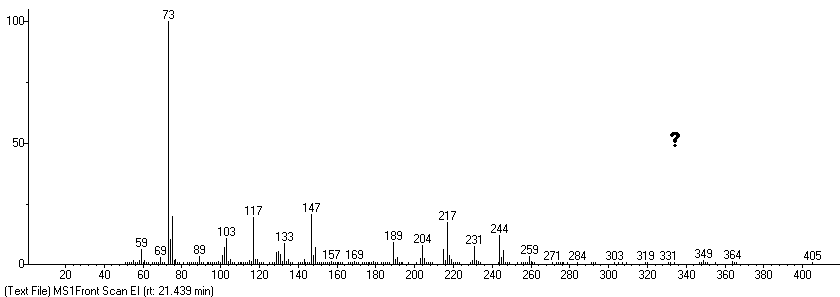

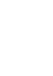


**Suppl 1. Fig. S103:** Mass spectrum of Arabinofuranose, 1,2,3,5- tetrakis-*O*-(trimethylsilyl), peak 9.


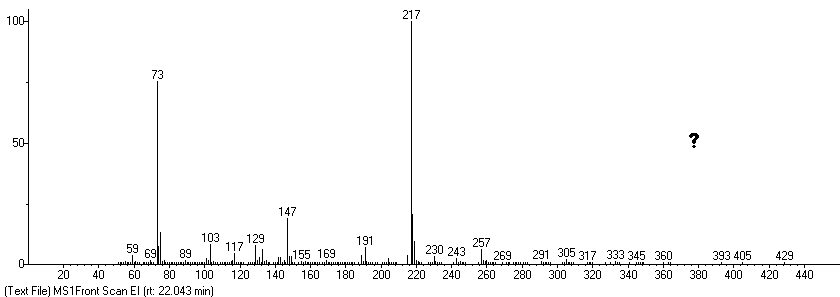

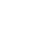


**Suppl 1. Fig. S104:** Mass spectrum of *D*-Allofuranose, pentakis (trimethylsilyl) ether, peak 10.


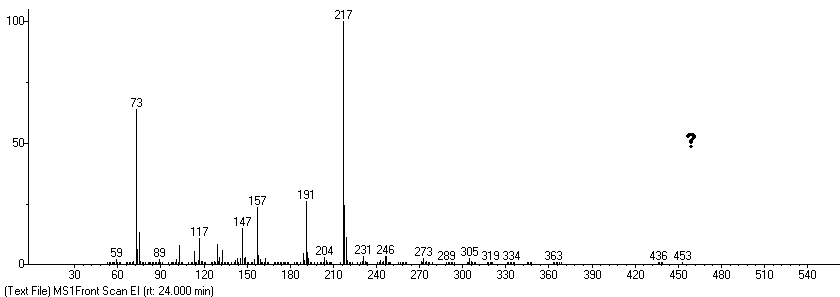

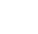


**Suppl 1. Fig. S105:** Mass spectrum of Methyl *α*-*D*- glucofuranoside, 4TMS, peak 11.


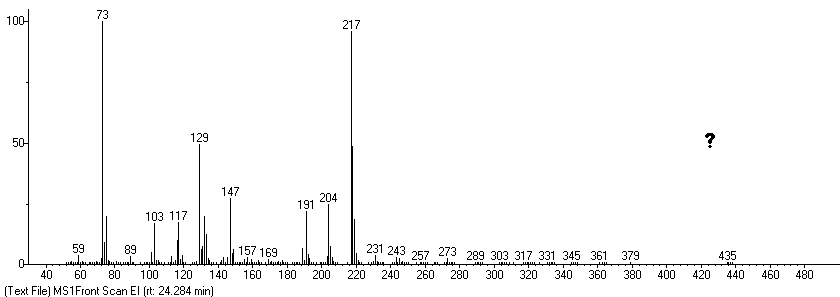

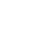


**Suppl 1. Fig. S106:** Mass spectrum of *D*-(-)-Fructofuranose, pentakis (trimethylsilyl) ether, peak 12.


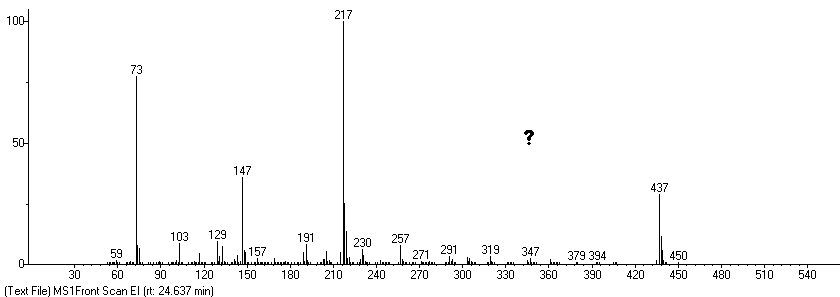

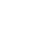


**Suppl 1. Fig. S107:** Mass spectrum of *D*-(+)-Talofuranose, pentakis (trimethylsilyl) ether, peak 13.


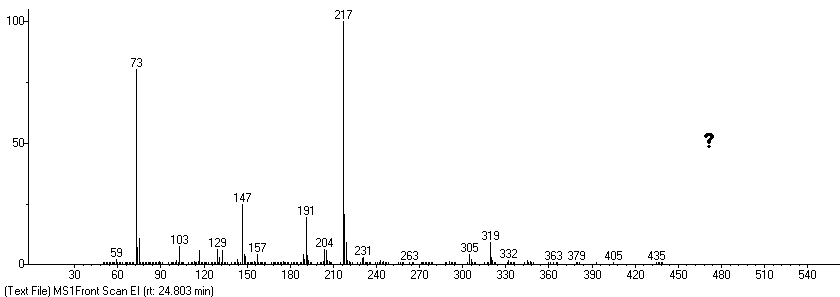

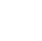


**Suppl 1. Fig. S108:** Mass spectrum of *D*-(-)-Ribofuranose, tetrakis (trimethylsilyl) ether, peak 14.


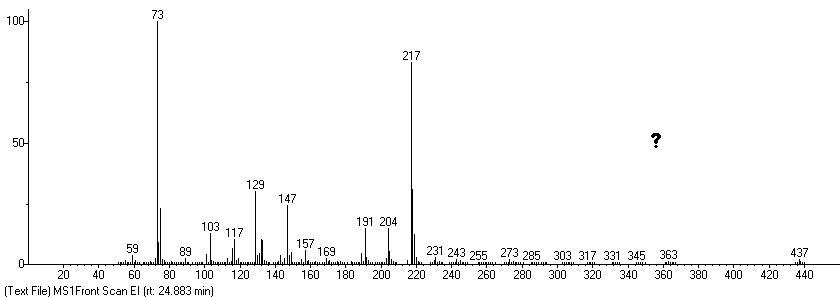

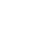


**Suppl 1. Fig. S109:** Mass spectrum of *β*-*D*-(+)-Talopyranose, 5TMS, peak 15.


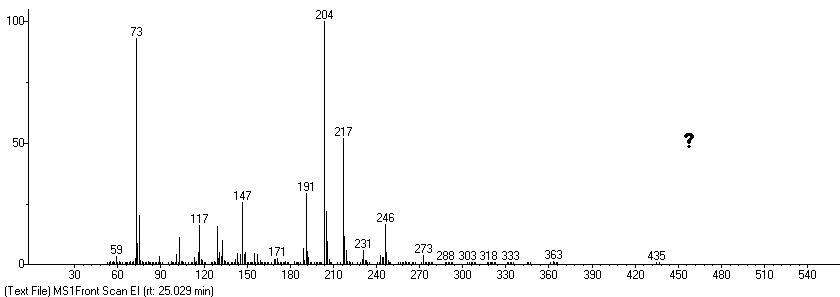

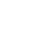


**Suppl 1. Fig. S110:** Mass spectrum of Quininic acid (5TMS), peak 16.


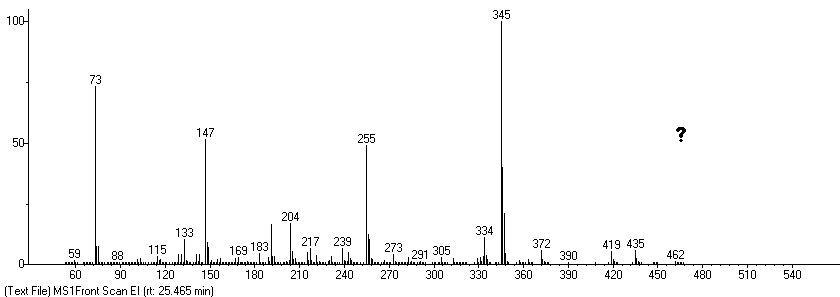

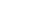


**Suppl 1. Fig. S111:** Mass spectrum of *L*-Glucono-1,4 lactone (2*S*,3*R*,4*S*,5*S*)-, 4TMS, peak 17.


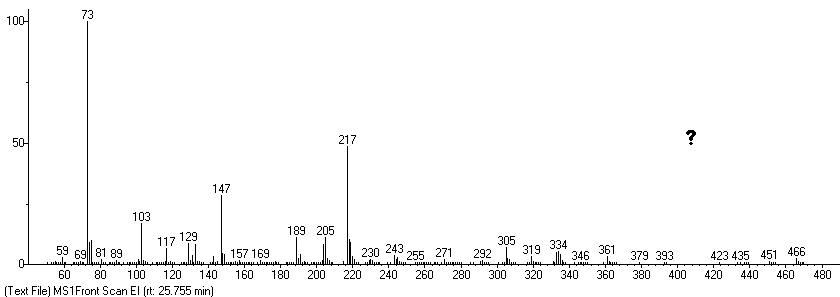

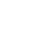


**Suppl 1. Fig. S112:** Mass spectrum of *β*-*D*-(+)-Mannopyranose, 5TMS, peak 18.


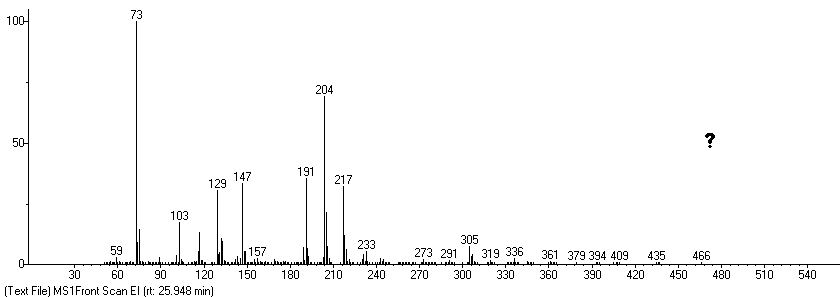

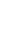


**Suppl 1. Fig. S113:** Mass spectrum of *α*-*D*-Allopyranose, 5TMS, peak 19.


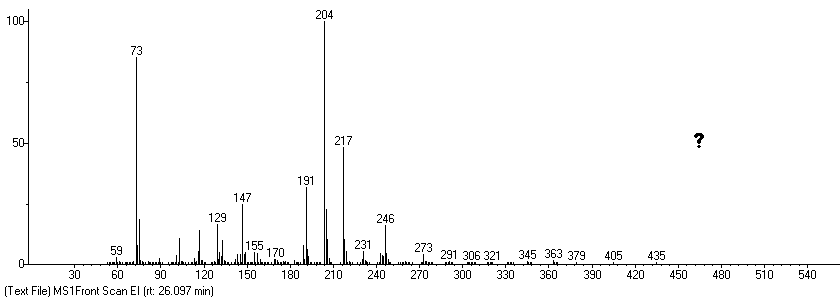

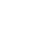


**Suppl 1. Fig. S114:** Mass spectrum of *D*-Altrose, 5TMS, peak 20.


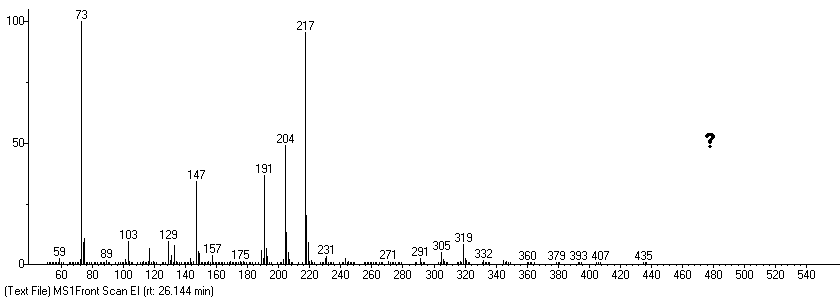

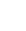


**Suppl 1. Fig. S115:** Mass spectrum of Gallic acid, 4TMS, peak 21.


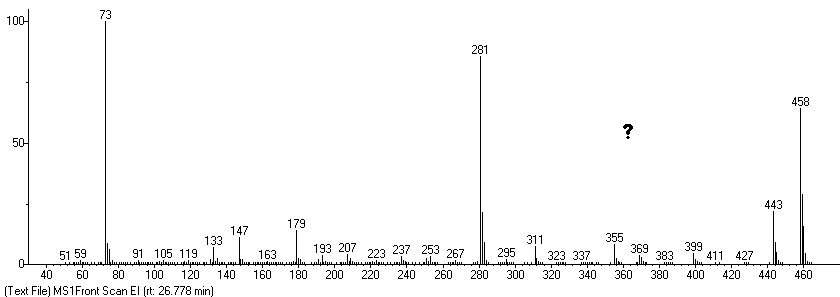

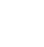


**Suppl 1. Fig. S116:** Mass spectrum of *β*-*D*-Allopyranose, 5TMS, peak 22.


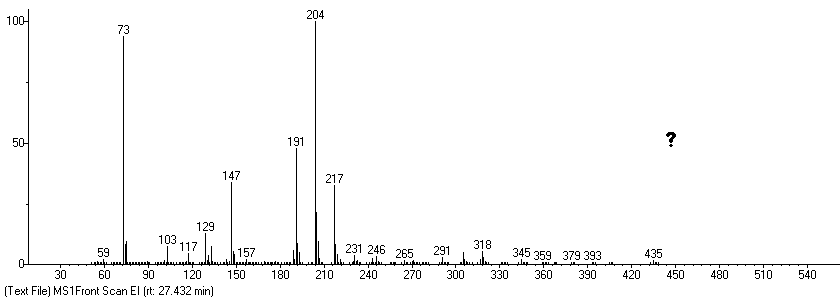

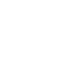


**Suppl 1. Fig. S117:** Mass spectrum of *D*-Gluconic acid, 6TMS, peak 23.


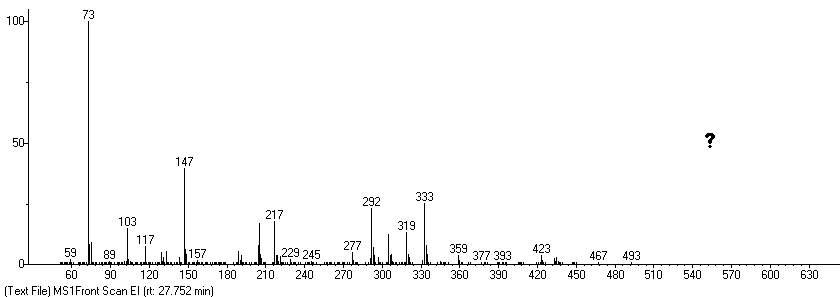

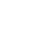


**Suppl 1. Fig. S118:** Mass spectrum of Palmitic Acid, TMS, peak 24.


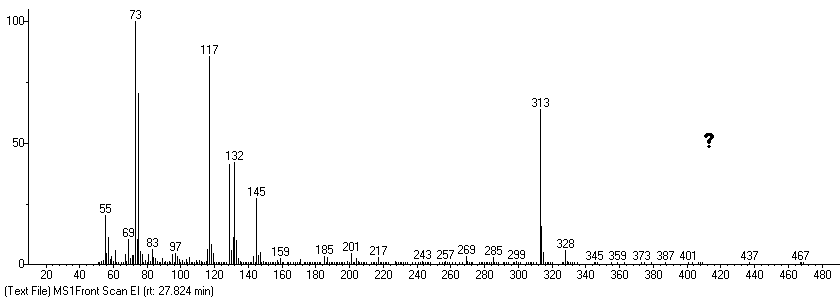

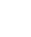


**Suppl 1. Fig. S119:** Mass spectrum of Sucrose, 8TMS, peak 25.


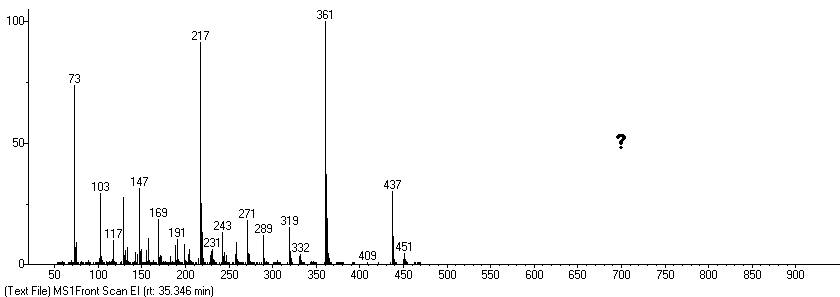

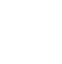


**Suppl 1. Fig. S120:** Mass spectrum of *D*-(+)-Turanose, octakis (trimethylsilyl) ether, peak 26.


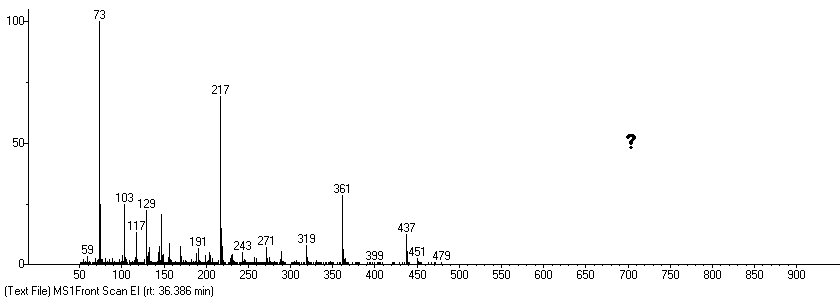

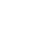


**Suppl 1. Fig. S121:** Mass spectrum of Lactose, 8TMS, peak 27.


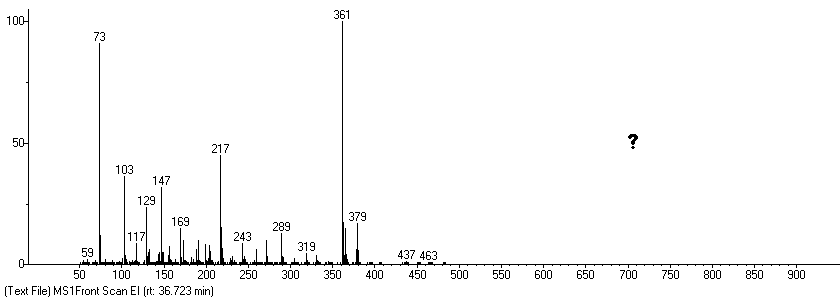

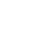


**Suppl 1. Fig. S122:** Mass spectrum of 3-*α*-Mannobiose, octakis (trimethylsilyl) ether, peak 28.


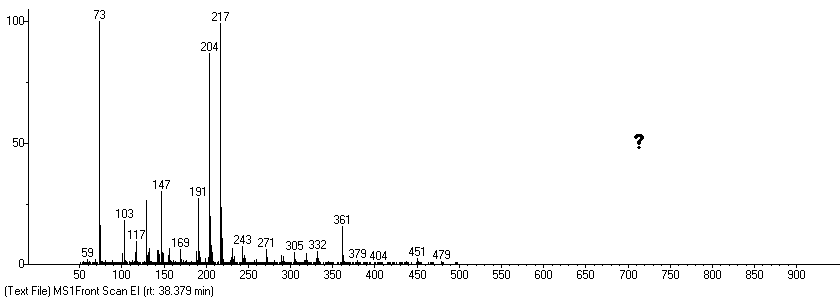

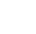


**Suppl 1. Fig. S123:** Mass spectrum of *D*-Lactose, 8TMS, peak 29.


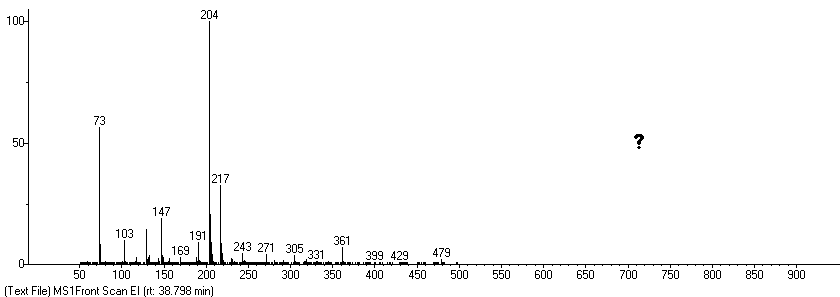

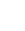


**Suppl 1. Fig. S124:** Mass spectrum of Galactinol, nonakis (trimethylsilyl) ether, peak 30.


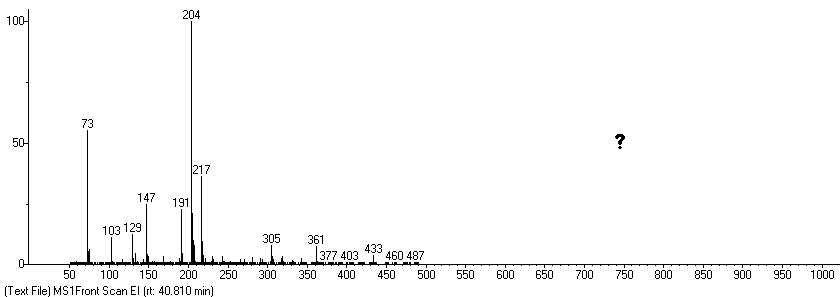

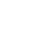


References

1. Alhalak N, Şen A, Öter GN, Akar RO, Ulukaya E, Şekerler T. The anti-angiogenic and cytotoxic potential of different Eremurus spectabilis fractions on hepatocellular carcinoma (Hep3B) cells: An in vitro, in Ovo, and in silico study. *Computers in Biology and Medicine*. 2025;194doi:10.1016/j.compbiomed.2025.110498

2. Ibrahim RM, Sedeek MS, Wareth AA, R. Khalifa M, Gendy AEM, Farag MA. Impact of cultivar types and thermal processing methods on sweet potato metabolome, a comparative analysis via a multiplex approach of NIR and GC–MS based metabolomics coupled with chemometrics. *Food Chemistry*. 2025;463doi:10.1016/j.foodchem.2024.141125

3. Fayek NM, Farag MA, Saber FR. Metabolome classification via GC/MS and UHPLC/MS of olive fruit varieties grown in Egypt reveal pickling process impact on their composition. *Food Chemistry*. 2021;339doi:10.1016/j.foodchem.2020.127861

4. Squara S, Caratti A, Fina A, et al. Artificial intelligence decision making tools in food metabolomics: Data fusion unravels synergies within the hazelnut (Corylus avellana L.) metabolome and improves quality prediction. *Food Research International*. 2024;194doi:10.1016/j.foodres.2024.114873

5. Rosso MC, Mazzucotelli M, Bicchi C, et al. Adding extra-dimensions to hazelnuts primary metabolome fingerprinting by comprehensive two-dimensional gas chromatography combined with time-of-flight mass spectrometry featuring tandem ionization: Insights on the aroma potential. *Journal of Chromatography A*. 2020;1614doi:10.1016/j.chroma.2019.460739

6. Yang C, Lu L, Liao L, et al. Establishment of GC–MS method for the determination of Pseudomonas aeruginosa biofilm and its application in metabolite enrichment analysis. *Journal of Chromatography B*. 2021;1179doi:10.1016/j.jchromb.2021.122839

7. Negi K, Asthana AK, Chaturvedi P. GC–MS analysis and antifungal activity of acetone extract of Conocephalum conicum (L) Underw (Liverwort) against aflatoxins producing fungi. *South African Journal of Botany*. 2020;131:384–390. doi:10.1016/j.sajb.2020.02.035

8. Öğüt K, Özek G, Öztürk N, Koray Yaylacı Ö, Özek T. Chemical composition, α-amylase inhibition, and antioxidant activities of Scabiosa hololeuca Bornm. *Biochemical Systematics and Ecology*. 2025;122doi:10.1016/j.bse.2025.105028
